# *α*-Synuclein regulates monoamine transporter capacity and amphetamine-induced efflux via modulation of transporter expression

**DOI:** 10.1016/j.jpet.2026.104981

**Published:** 2026-07-04

**Authors:** Iina Ludwig, Samuel Moser, Marina Lukenic, Michael Connor, Amila Sofic, Samantha Kaltenbrunner, Constantin Pätzold, Alexander Hausleitner, Catherine Gilbert, Sonja Sucic, Marko Roblek, Thomas Steinkellner

**Affiliations:** Institute of Pharmacology, Center for Physiology and Pharmacology, Medical University of Vienna, Vienna, Austria

**Keywords:** *α*-Synuclein, Monoamine transporters, Parkinson disease, Dopamine, Serotonin, Amphetamine

## Abstract

*α*-Synuclein (*α*-syn) is a presynaptic protein implicated in Parkinson disease and related synucleinopathies, but its physiological function remains incompletely understood. Monoamine transporters, including the dopamine transporter (DAT) and serotonin transporter (SERT), are key determinants of neurotransmitter clearance and primary targets of psychostimulant drugs. Although *α*-syn has been reported to interact with these transporters, its impact on transporter function remains unresolved. In the present study, we systematically manipulated *α*-syn expression in cellular models to determine its effects on DAT and SERT function. Overexpression of *α*-syn increased substrate uptake at both transporters, reflected by a selective increase in maximal transport velocity (V_max_) without significant changes in substrate affinity (K_M_). This effect was accompanied by elevated total DAT and SERT protein levels. Conversely, small interfering RNA-mediated knockdown or CRISPR/Cas9-mediated knockout of *α*-syn reduced transporter expression and decreased uptake capacity. In addition, amphetamine-induced transporter-mediated efflux was significantly attenuated in *α*-syn-deficient cells. These findings identify *α*-syn as a positive regulator of monoamine transporter expression and transport capacity. By controlling transporter abundance, *α*-syn modulates both substrate uptake and psychostimulant-induced reverse transport. This regulatory mechanism has important implications for monoamine homeostasis and for the pharmacological actions of transporter-targeting drugs such as D-amphetamine or 3,4-methylenedioxymethamphetamine (“ecstasy”).

**Significance Statement:**

The physiological role of *α*-synuclein (*α*-syn), a protein central to Parkinson disease, is not fully understood. This study demonstrates that *α*-syn enhances the expression and transport capacity of dopamine and serotonin transporters, thereby regulating monoamine clearance and amphetamine-induced efflux. This identifies *α*-syn as a key modulator of monoaminergic signaling and provides a mechanistic framework linking its normal function to disease-related alterations in neurotransmission.

## 1 Introduction

The small protein *α*-synuclein (*α*-syn) was identified in the late 1990s as a major constituent of Lewy bodies in postmortem brains of patients with Parkinson disease (PD).^1^ Around the same time, missense mutations in the *α*-synuclein gene (*SNCA; PARK4*) were shown to cause autosomal-dominant PD.^2^ Despite extensive investigation since then, the physiological function of *α*-syn remains incompletely understood.^3^

*α*-Syn is highly enriched at presynaptic terminals and has been implicated in vesicular trafficking and neurotransmitter release.^4^^,^^5^ However, genetic deletion of *SNCA* in mice results in only subtle alterations in transmitter release, raising questions about its precise physiological role.^6^

In addition to its proposed role in synaptic vesicle dynamics, *α*-syn has been reported to interact directly with the dopamine (DA) transporter (DAT; *SLC6A3*).^7^ DAT is expressed in dopaminergic neurons, the neuronal population most prominently affected in PD, and plays a central role in DA homeostasis and as a target for psychostimulants such as amphetamines and cocaine. Thus, an interaction between *α*-syn and DAT may be of both physiological and pharmacological relevance. Moreover, *α*-syn has also been shown to interact with the closely related norepinephrine (NET; *SLC6A2*) and serotonin (SERT; *SLC6A4*) transporters,^8^^,^^9^ the major targets of most clinically used antidepressants.

Despite these observations, the functional consequences of *α*-syn interactions with monoamine transporters remain unclear. Initial studies reported increased DA uptake upon coexpression of *α*-syn and human DAT without changes in substrate affinity.^7^

In contrast, other studies described reduced DAT mediated uptake in various cell models.^10^ Subsequent work has reported either increased or decreased transporter activity after *α*-syn overexpression in cells and in vivo models.^11^, ^12^, ^13^, ^14^ Functional effects of *α*-syn on SERT and NET have been less extensively studied but have generally suggested reduced substrate uptake.^8^^,^^9^ These discrepancies may reflect differences in experimental systems, expression levels, or *α*-syn conformational states.

Unlike prior studies that examined individual manipulations of *α*-syn or single transporters, our work systematically compares overexpression, knockdown, and knockout effects on both DAT and SERT, providing a comprehensive assessment of *α*-syn-dependent regulation. Our findings provide new insight into the role of *α*-syn in regulating monoamine transporter capacity and its consequences for psychostimulant responses.

## 2 Materials and methods

### 2.1 Materials

3,4-[Ring-2,5,6-^3^H]-dihydroxyphenylethylamine (^3^H-dopamine; 1 mCi mL^−1^) and 5-[1,2-^3^H(N)]-hydroxytryptamine creatinine sulfate (^3^H-serotonin; 5-hydroxytryptamine [5-HT]; 1 mCi/mL^−1^) were purchased from Perkin-Elmer/Revvity. 1-[Methyl-^3^H]-4-phenylpyridinium iodide (^3^H-MPP^+^; 1 mCi/mL^−1^) was purchased from Hartmann Analytic GmbH. Scintillation fluid (Rotiszint eco plus) was purchased from Carl Roth GmbH. D-amphetamine, 3,4-methylenedioxymethamphetamine (MDMA), GBR12909, mazindol and paroxetine were from Sigma-Aldrich. Details of other materials and their suppliers are provided in the specific subsections.

### 2.2 Animals

Mice were used in accordance with protocols approved by the Animal Welfare Committee of the Medical University of Vienna and the Austrian Federal Ministry of Science and Research (BMFWF licenses 2021-0.373.073 and 2022-0.920.727).

### 2.3 Cell culture

Neuro2a (N2a) cells, Cath.a-differentiated (CAD) and human embryonic kidney 293 (HEK-293) cells are from ATCC. N2a and HEK-293 cells were cultured in Dulbecco’s modified Eagle’s medium, 10% fetal calf serum and 100 U/mL penicillin and 100 *μ*g/mL streptomycin. CAD cells were cultured in 50% Dulbecco’s modified Eagle’s medium/50% Ham’s F12 medium supplemented with 10% fetal calf serum and 100 U/mL penicillin and 100 *μ*g/mL streptomycin. Cells were transfected with jetPRIME (Polyplus) or Lipofectamine 2000 (Invitrogen) according to the manufacturer’s instructions with the following plasmids: enhanced yellow fluorescent protein (eYFP)-N-terminally tagged wild-type human DAT or SERT (in pcDNA3.1), “empty” vector (pcDNA3.1). Generation of wild-type human *α*-syn in a pAAV-DIO-hSyn1 backbone has been described.^15^ Note, that the *α*-syn plasmid used for transfections was in the “Cre-off” configuration, that is, expression is not dependent on Cre recombinase expression. For *α*-syn overexpression experiments, transporter plasmids were combined with human *α*-syn plasmid or empty vector in a 1:3 ratio in uptake/efflux assays or at the ratios indicated in the dose-response uptake experiment.

### 2.4 Small interfering RNA knockdown of human *α*-syn in HEK-293 cells

HEK-293 cells were seeded onto poly-D-lysine-coated 48-well plates. On the next day, cells were transfected with a mix of 3 different human *SNCA* small interfering RNAs (siRNAs) (20 *μ*M, Invitrogen Cat Number: 10620318 and 10620319), with the following sequences: SNCAHSS144006: GGGAGUUGUGGCUGCUGCUGAGAAA/UUUCUCAGCAGCAGCCACAACUCCC, SNCAHSS186008: GACCAAAGAGCAAGUGACAAAUGUU/AACAUUUGUCACUU GCUCUUUGGUC, SNCAHSS144005: UCAAGACUACGAACCUGAA GCCUAA/UUAGGCUUCAGGUUCGUAGUCUUGA.

Control cells were transfected with Stealth RNAi siRNA Negative Control (20 *μ*M, Invitrogen, #452001). Lipofectamine RNAiMAX reagent (Invitrogen, #13778-150) was used for siRNA transfections according to the manufacturer’s instructions. The final siRNA concentration on the cells was 10 nM. siRNAs were prepared in Opti-MEM Reduced Serum Medium (Gibco, #11058-021). The cells were incubated for 48 hours, before transfections with plasmids encoding eYFP-hDAT or eYFP-hSERT using Lipofectamine 2000 (Invitrogen, #11668-019). The cells were incubated for an additional 24 hours before performing uptake assays.

### 2.5 CRISPR/Cas9 knockout of human SNCA in HEK-293 cells

Predesigned human *SCNA* specific crRNA targeting exon 4 of human *SNCA* (5′-GTCCTTTTTGACAAAGCCAG-3′; PAM: TGG; ALT-R Hs.Cas9.SNCA.1.AA CRISPR-Cas9 crRNA, Integrated DNA Technologies) was mixed in equimolar ratio with transactivating CRISPR RNA (ALT-R CRISPR-Cas9 tracrRNA, Integrated DNA Technologies) in IDTE buffer (pH = 8, 10 mM Tris, 0.1 mM EDTA) to yield a 100 *μ*M stock. The crRNA/tracrRNA mix was diluted to 1 *μ*M in Nuclease Free Duplex Buffer (Integrated DNA Technologies), incubated at 95 °C for 5 minutes and cooled down on a heating block to 25 °C to enable duplex formation.

HEK-293 cells were cultured in antibiotic-free medium and seeded onto a 24-well plate to reach 70% confluence the following day. Cells were transfected using Lipofectamine CRISPRMAX (Invitrogen, #CMAX00008) according to the manufacturer’s instructions. In detail, per 24-well 10 pmol of SpCas9 nuclease (Integrated DNA Technologies, #1081058), 10 pmol of previously prepared cr/tracrRNA duplex, and 2 *μ*L of the CRISPRMAX reagent were used. One day after transfection, cells were transferred to a 6 cm expansion dish. Three days after transfection, cells were single-cell cloned by limited dilution into 96-well plates. Two weeks later, single-cell colonies were selected by visual inspection using an Olympus CKX41 microscope with an Olympus Plan C N 4×/0.10 objective (Olympus) and expanded onto 24-well plates. A total of 53 suitable looking clones was then expanded to 6-well plates, of which 38 were lysed using radioimmunoprecipitation assay (RIPA) buffer and were screened by immunoblot analysis. Four possible *α*-syn-knockout candidates were chosen based on the *α*-syn immunoblot result. A clone with intact *α*-syn expression was picked as a wild-type control. Picked clones were propagated for further experiments. The SCNA^−/−^ cell line was sequenced to confirm genomic *SNCA* knockout.

### 2.6 Uptake assays

Experiments were performed as previously described.^16^ One day before the experiment, cells expressing the respective transporter were seeded onto poly-D-lysine-coated 48 well plates. For uptake experiments, the cell culture medium was replaced with 100 *μ*L Krebs-HEPES buffer (KHB; 10 mM HEPES, 120 mM NaCl, 3 mM KCl, 2 mM CaCl_2_.2H_2_O, 2 mM MgCl_2_.6H_2_O, 20 mM D-(+)-glucose-monohydrate, pH = 7.3) at room temperature. The buffer was replaced with 0.1 *μ*M [^3^H]-dopamine (DAT), 0.1 *μ*M [^3^H]-5-HT (SERT), or 0.05 *μ*M [^3^H]-MPP^+^ (DAT) and various concentrations of unlabeled substrate in a total volume of 100 *μ*L KHB. Uptake was terminated after 2 minutes (DA), 1 minute (5-HT), or 3 minutes (MPP^+^) by washing the cells with 250 *μ*L ice-cold KHB. Subsequently, the cells were lysed with 200 *μ*L 1% SDS, added to scintillation vials containing liquid scintillation cocktail, and the amount of ^3^H in the cells was measured using a liquid scintillation counter. Nonspecific uptake was determined in the presence of saturating antagonist concentrations, that is, 10 *μ*M mazindol or 1 *μ*M GBR12909 (for DAT) or 10 *μ*M paroxetine (for SERT). Uptake data were fitted by nonlinear regression, V_max_ and K_M_ values were calculated from the Michaelis–Menten equation with GraphPad Prism.

After siRNA-mediated knockdown, a radioligand uptake assay was performed using either [^3^H]-MPP^+^, [^3^H]-dopamine or [^3^H]-serotonin. Three wells of both knockdown and control were used to measure specific uptake and another 3 wells each to measure nonspecific uptake. Specific uptake reaction was initiated by adding KHB containing either 0.05 *μ*M [^3^H]-MPP^+^ or 0.1 *μ*M [^3^H]-dopamine or 0.1 *μ*M [^3^H]-serotonin to the wells in 10 second intervals and incubated at room temperature for either 3 minutes (MPP^+^), 2 minutes (dopamine), or 1 minute (serotonin). After incubation, the reaction was rapidly stopped by removing the radioactive buffer and washing 3 times with ice-cold KHB. Preincubation for nonspecific uptake wells was done by adding 10 *μ*M mazindol for DAT or 10 *μ*M paroxetine for SERT (10 *μ*M each) for 5 minutes.

### 2.7 Release assay

Release experiments are described in detail in Steinkellner et al.^17^ Cells were transfected using Lipofectamine 3000 with either DAT or SERT and/or *α*-syn or empty vector and incubated with 0.05 *μ*M [^3^H]-MPP^+^ (for DAT) or 0.1 *μ*M [^3^H]-5-HT (for SERT) in KHB for 20 minutes at 37 °C. Subsequently, cells were placed in superfusion chambers that were perfused for 40 minutes at a flow rate of 0.5 mL/min (25 °C) to establish a stable basal efflux of radioactivity. The end of this washout period corresponded to the zero-time point. The superfusate was collected in intervals of 2 minutes, resulting in fractions of 1 mL. After 6 minutes, 10 *μ*M D-amphetamine (for DAT) or 10 *μ*M MDMA (for SERT) were added to KHB to induce efflux. The superfusion with D-amphetamine or MDMA lasted for 10 minutes, resulting in the collection of 5 fractions; thereafter, cells were lysed in 1% SDS, and mixed with scintillation mixture to determine the total amount of radioactivity present at the end of the experiment. Efflux of radioactive substrate is expressed as percentage of total radioactivity (ie, the sum of the total remaining radioactivity and the released radioactivity) present at each time point.

### 2.8 Immunoblotting

Whole-cell lysates were prepared using RIPA lysis buffer (150 mM NaCl, 10 mM EDTA, 0.1% SDS, 0.1% Triton X-100, 0.5% sodium deoxycholate, 50 mM Tris-HCl, pH = 7.4). Cells were first grown in plastic dishes at 37 °C, put on ice and washed twice using ice-cold phosphate-buffered saline (2.7 mM KCl, 1.5 mM KH_2_PO_4_, 137 mM NaCl, 4.3 mM Na_2_HPO_4_.2H_2_O, pH = 7.3). RIPA buffer supplemented with protease inhibitors (Roche complete) was then added to lyse the cells. The cells were scraped from the plate and collected in plastic tubes. The homogenates were further incubated for 30 minutes on a rotator at 4 °C, after which they were centrifuged at 14,000 rpm for 10 minutes at 4 °C. The supernatant was collected and kept at −20 °C until further use. The protein concentrations were determined using the BCA method (Pierce BCA Protein Assay Kit, Thermo Fisher, #23225).

To detect *α*-syn in mouse striatum, a male wild-type C57BL6/J mouse was anesthetized with pentobarbital (100 mg/kg, i.p.; Exagon 500 mg/mL; Richter Pharma) and killed by cervical dislocation, brain was removed and striatum dissected. Striatum was homogenized in RIPA buffer as described above for cells.

For immunoblotting, Laemmli sample buffer (0.0625 M Tris-HCl, pH = 6.8, 2% SDS, 10% glycerol, 0.01% bromophenol blue, and 5% beta-mercaptoethanol) was added to samples (1:6), and samples were incubated for either 5 minutes in 95 °C (for detection of *α*-syn) or 30 minutes at 37 °C (for detecting DAT and SERT). Proteins were separated using either 10% (DAT and SERT) or 12% (*α*-syn) SDS-polyacrylamide gels and separated by electrophoresis in SDS running buffer (25 mM Tris, 192 mM Glycine, 0.1 mM EDTA.Na_2_, 0.175mM SDS, pH = 8.5) at 180 V. Transfer onto nitrocellulose membranes was done by wet transfer (transfer buffer: 25 mM Tris, 192 mM Glycine, 10% methanol, pH = 8.3) at 80 V for 45 minutes. For detection of *α*-syn the transfer was done onto methanol-activated polyvinylidene difluoride membranes at 250 mA for 1.5 hours and membranes were fixed after transfer for 30 minutes in 0.4% paraformaldehyde at room temperature to increase the binding of monomeric *α*-syn to the membrane.^18^ Membranes were then stained with Ponceau S (0.1% Ponceau S [w/v] in 5% acetic acid [v/v]) for about 1 minute to visualize protein loading and transfer efficiency followed by blocking for 1 hour in 5% nonfat dry milk in Tris-buffered saline-Tween-20 (20 mM Tris, 150 mM NaCl, 0.1% Tween-20, pH = 7.5). After blocking, membranes were incubated in primary antibody solution overnight at 4 °C. Primary antibodies were diluted in blocking buffer: mouse anti-*α*-syn 4D6 (BioLegend, #834301), 1:1000; rat anti-human-*α*-syn 15G7 (Enzo ALX-804-258-L001), 1:100; rabbit anti-green-fluorescent protein (Invitrogen, Thermo Fisher Scientific #A11122), 1:2000; rabbit anti-glyceraldehyde-3-phosphate dehydrogenase (GAPDH) (D16H11) XP (Cell Signaling Technologies, #5174S), 1:5000. After primary antibody incubation, membranes were washed 3 times for 5 minutes with Tris-buffered saline-Tween-20, after which they were incubated with secondary antibodies (in blocking buffer at a concentration of 1:5000) for 2 hours in room temperature. The secondary antibodies used were IRDye goat anti-rabbit 800 (926-32213), goat anti-rabbit 680 (#926-68071), and goat anti-mouse 680 (#926-68070) (LI-COR Biotechnology). After secondary antibody incubation membranes were washed 3 times for 5 minutes with Tris-buffered saline-Tween-20 and then for 5 minutes with Tris-buffered saline. Membranes were imaged using a LI-COR Odyssey CLx imaging system and analyzed as described below.

### 2.9 Data analysis and statistics

Uptake and release assays were performed in triplicate measurements. For analysis of uptake assays, the means of the counts per minute were corrected by subtracting the background (= the means of the counts from the nonspecific uptake, ie, the uptake in the presence of a specific inhibitor). Counts per minute were then transformed to pmol/million cells/min. Nonlinear regression analysis was performed using GraphPad Prism. To determine kinetic parameters K_M_ and V_max_, the Michaelis–Menten equation was fitted to the data.

Immunoblot images were analyzed using Fiji (ImageJ) to quantify the intensity of the protein bands. Briefly, regions of interest were manually delineated using the region of interest manager tool. Pixel intensity measurements within these regions of interest were obtained and mean intensity values were calculated. GAPDH was used as a loading control. Protein amounts were quantified as the ratio between the protein of interest divided by the GAPDH loading control band.

For all tests, *P* < .05 was considered statistically significant; this is indicated by a star (∗) in the respective figures. Details for all tests calculated can be found in the figure legends.

Two-tailed paired *t* tests were used for comparisons between 2 matched groups, whereas comparisons among more than 2 matched conditions were analyzed using the nonparametric Friedman test because of the repeated measures design and small sample size. For the release assay, a two-way ANOVA was used with time and treatment as factors.

GraphPad Prism 10 (GraphPad Software Inc; RRID:SCR_002798) was used to analyze data and create graphs. All data are expressed as mean ± SEM. All “*n*” reported (number of observations) refer to biological replicates.

## 3 Results

### 3.1 Overexpression of *α*-syn increases DAT and SERT uptake rates in N2a cells

To examine the effect of *α*-syn on monoamine transporter function, we overexpressed *α*-syn together with human DAT or SERT in N2a cells, a mouse neuroblastoma cell line. N2a cells were transfected with eYFP-tagged DAT or SERT and either human wild-type *α*-syn or control plasmid (pcDNA3.1) **(**Fig. 1**)**. Radiolabeled substrate uptake was measured 48 hours after transfection.

**Fig. 1:**
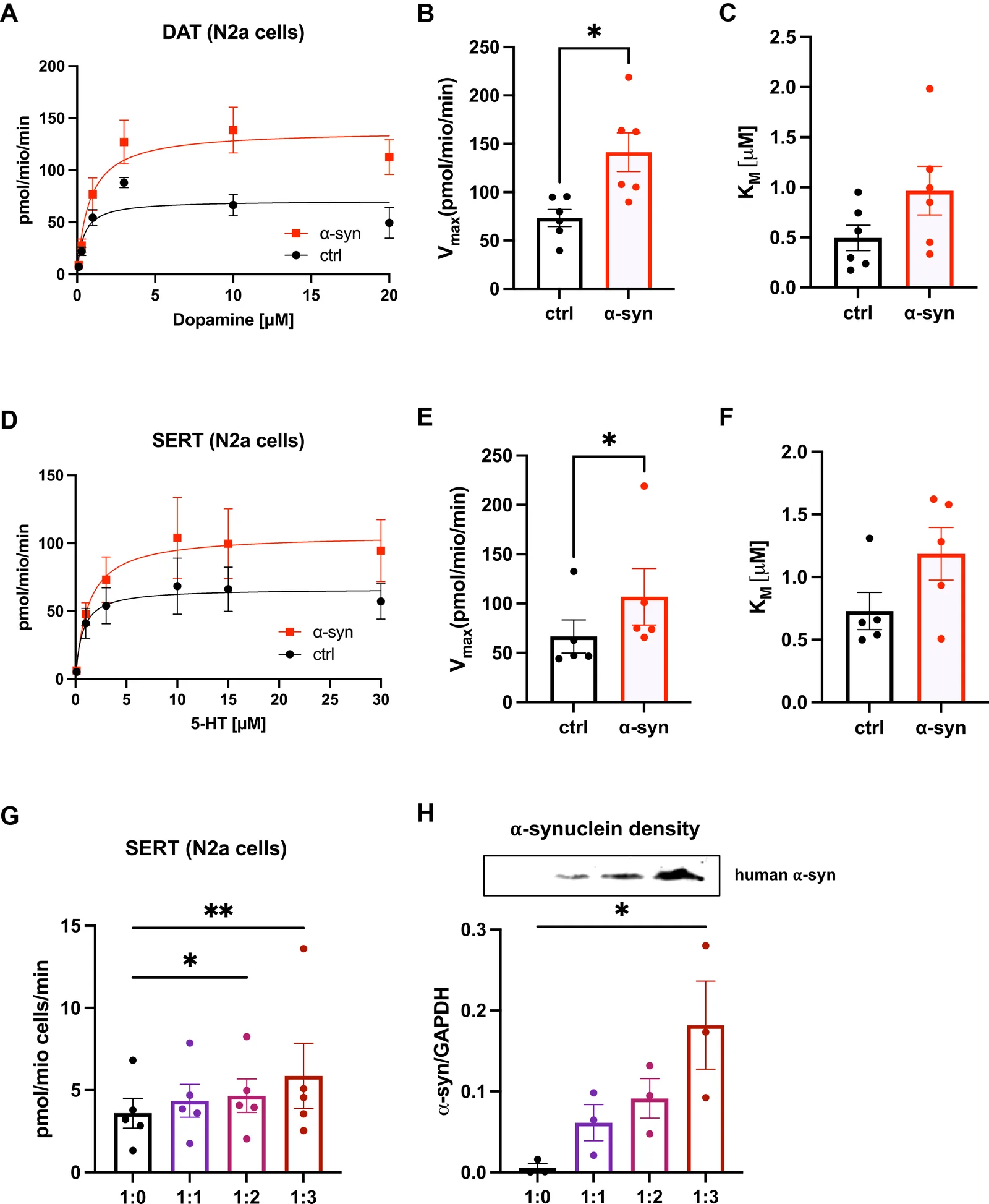
Overexpression of *α*-syn in N2a cells increases DAT and SERT uptake. (A) Michaelis–Menten kinetics of DA uptake in N2a cells transfected with human DAT and pcDNA3.1 (control) or *α*-syn. (B) V_max_ for DA uptake at DAT: ctrl V_max_: 73.4 ± 8.78 pmol/mio cells/min (*n* = 6); *α*-syn: 141.4 ± 20.01 pmol/mio cells/min (*n* = 6); two-tailed paired *t* test: *t* = 3.467, df = 5, ∗*P* = .0179. (C) K_M_ for DA at DAT: ctrl: K_M_ = 0.5 ± 0.13 *μ*M; *α*-syn: K_M_ = 0.9 ± 0.24 *μ*M (*n* = 6); two-tailed paired *t* test: *t* = 0.0773, df = 5, *P* = .0773. (D) Michaelis–Menten kinetics of 5-HT uptake in N2a cells transfected with human SERT and pcDNA3.1 (control) or *α*-syn. (E) V_max_ for 5-HT uptake at SERT: ctrl V_max_: 66.7 ± 16.76 pmol/mio cells/min (*n* = 5); *α*-syn: 106.9 ± 28.65 pmol/mio cells/min (*n* = 5); two-tailed paired *t* test: *t* = 2.946, df = 4, ∗*P* = .0422. (F) K_M_ for 5-HT at SERT: ctrl: K_M_ = 0.7 ± 0.15 *μ*M; *α*-syn: K_M_ = 1.2 ± 0.21 *μ*M; two-tailed paired *t* test: *t* = 2.118, df = 4, *P* = .1016. (G) Uptake of [^3^H]-5HT (5HT = serotonin; 0.1 *μ*M) in N2a cells transiently transfected with different SERT/*α*-syn plasmid ratios: “1:0” (1 part SERT, 3 parts empty vector), “1:1” (1 part SERT, 1 part *α*-syn, 2 parts empty vector), “1:2” (1 part SERT, 2 parts *α*-syn, 1 part empty vector), “1:3” (1 part SERT, 3 parts *α*-syn). Uptake rate: “1:0”: 3.6 ± 0.90 pmol/min/mio cells; “1:1”: 4.4 ± 1.00 pmol/min/mio cells; “1:2”: 4.7 ± 1.02 pmol/min/mio cells; “1:3”: 5.9 ± 1.98 pmol/min/mio cells. Friedman test: ∗∗*P* = .0014; Dunn’s multiple comparisons test: 1:0 vs 1:2: ∗*P* = .0423; 1:0 vs 1:3: ∗∗*P* = .0087. *n* = 5/group. (H) Densitometric analysis of *α*-syn bands detected using the 15G7 antibody: Upper panel: representative immunoblot. Lower panel: quantification of *α*-syn (normalized to GAPDH): “1:0”: 0.006 ± 0.0051; “1:1”: 0.061 ± 0.0224; “1:1”: 0.091 ± 0.0244. “1:3”: 0.182 ± 0.0544. Friedman test: ∗*P* = .0174; Dunn’s multiple comparisons test: 1:0 vs 1:3: ∗*P* = .0342. *n* = 3/group.

Coexpression of *α*-syn significantly increased the maximal uptake velocity (V_max_) of both DAT (Fig. 1, A–C) and SERT (Fig. 1, D–F). In contrast, substrate affinity (K_M_) was not significantly altered for either transporter.

We next tested whether uptake velocity was directly related to *α*-syn expression levels by varying the amount of *α*-syn plasmid while keeping the amount of transporter plasmid constant. In N2a cells transfected with hSERT, 5-HT uptake increased proportionally with increasing *α*-syn plasmid concentrations, supporting a direct relationship between *α*-syn expression levels and transport velocity (Fig. 1, G and H; Supplemental Fig. 1).

### 3.2 N2a cells express low endogenous levels of *α*-syn

To assess endogenous *α*-syn expression in N2a cells, we compared *α*-syn protein levels in N2a, murine CAD and HEK-293 cells with *α*-syn from mouse striatum by immunoblotting. HEK-293 cells are commonly used for heterologous protein expression, whereas CAD cells are catecholaminergic and express several neuronal markers.^19^

*α*-Syn protein levels were readily detectable in HEK-293 cells but were barely detectable in N2a and CAD cells (Fig. 2), which is also in line with previous findings.^18^ Based on these observations, HEK-293 cells were selected for subsequent experiments examining the effects of endogenous *α*-syn on monoamine transporter function.

**Fig. 2:**
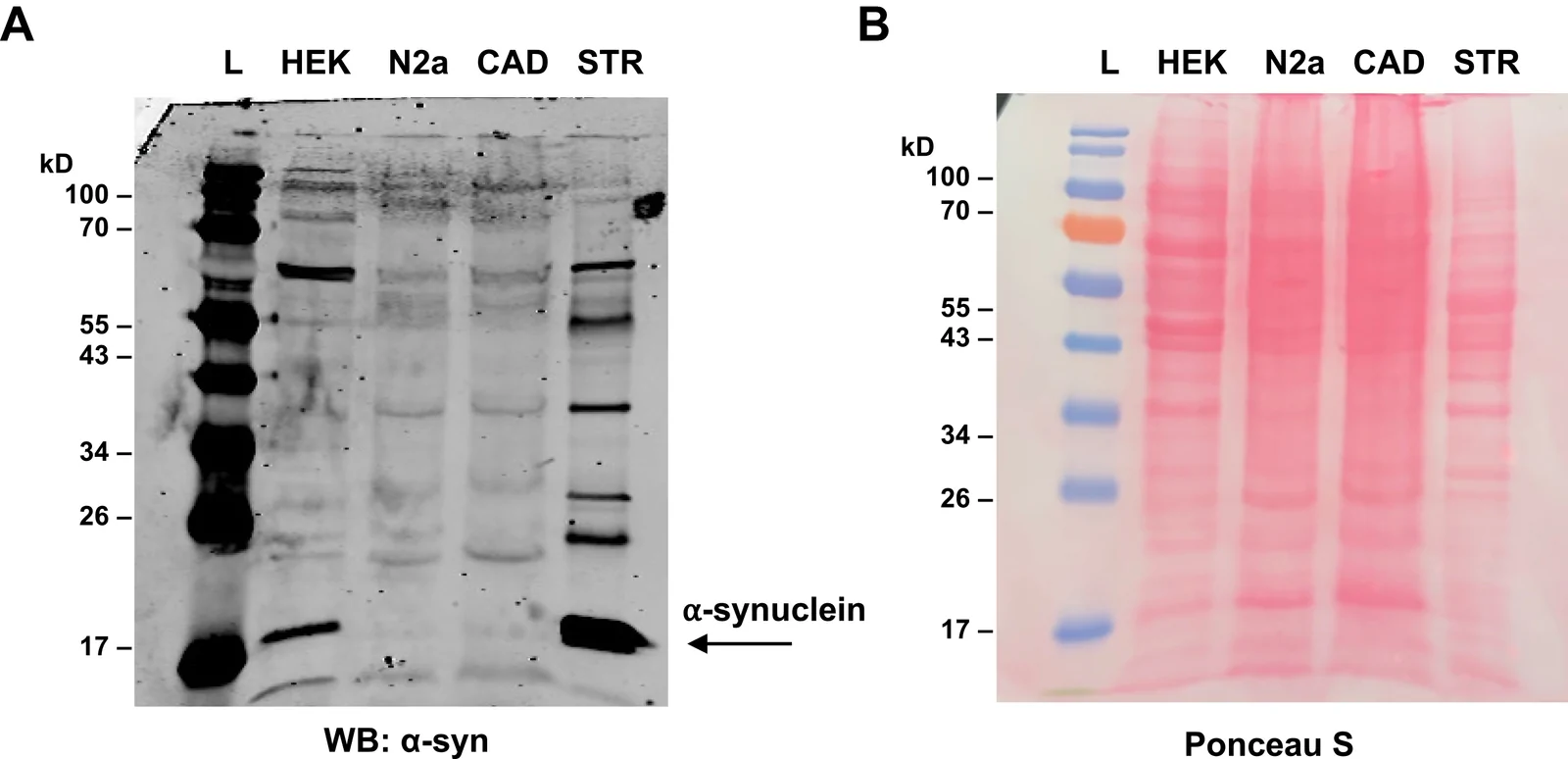
*α*-Syn is abundantly expressed in HEK-293 but not N2a or CAD cells. (A) Immunoblot of *α*-syn expression using the 4D6 antibody in HEK-293 (“HEK”), Neuro2a (“N2a”) and CAD cells (“CAD”); mouse striatum (“STR”) was loaded as positive control. (B) Ponceau S staining of membrane is shown as loading control. *L*, protein ladder.

### 3.3 Overexpression of *α*-syn increases DAT and SERT uptake rates in HEK-293 cells

To determine whether the effects observed in N2a cells were reproducible in a second cell system, we overexpressed *α*-syn together with DAT or SERT in HEK-293 cells and performed uptake assays 48 hours after transfection.

Consistent with the N2a cell data, *α*-syn overexpression in HEK-293 cells significantly increased uptake of 2 different substrate, [^3^H]-DA and [^3^H]-MPP^+^, at DAT as reflected by increased V_max_ values (Fig. 3, A and B; Supplemental Fig. 2, A and B). Similarly, V_max_ increased for [^3^H]-5-HT (= serotonin) uptake at SERT (Fig. 3, D–F). Again, K_M_ values were not significantly altered for any of the substrates (Fig. 3, C and F; Supplemental Fig. 2C).

**Fig. 3:**
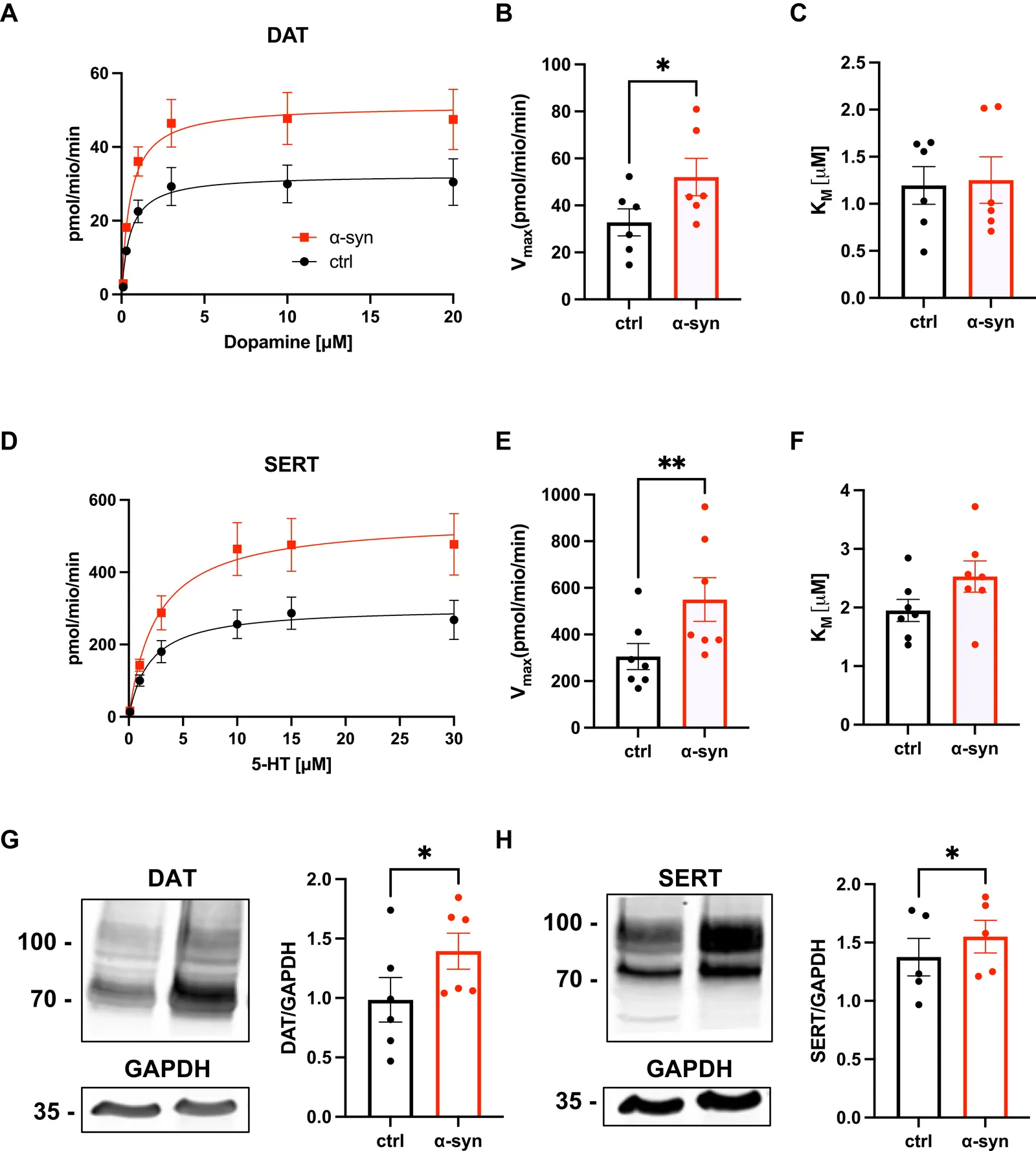
Overexpression of *α*-syn in HEK-293 cells increases DAT and SERT uptake. (A) Michaelis–Menten kinetics of DA uptake in HEK-293 cells transfected with human DAT and pcDNA3.1 (control) or *α*-syn. (B) V_max_ for DA uptake at DAT: ctrl V_max_: 32.8 ± 5.74 pmol/mio cells/min (*n* = 6); *α*-syn: 52.1 ± 7.98 pmol/mio cells/min (*n* = 6); two-tailed paired *t* test: *t* = 3.604, df = 5, ∗*P* = .0155. (C) K_M_ for DA at DAT: ctrl: K_M_ = 1.2 ± 0.20 *μ*M (*n* = 6); *α*-syn: K_M_ = 1.3 ± 0.24 *μ*M (*n* = 6); two-tailed paired *t* test: *t* = 0.3818, df = 5, *P* = .7183. (D) Michaelis–Menten kinetics of 5-HT uptake in HEK-293 cells transfected with human SERT and pcDNA3.1 (control) or *α*-syn. (E) V_max_ for 5-HT uptake at SERT: V_max_ for 5-HT uptake at SERT: ctrl V_max_: 305.4 ± 55.56 pmol/mio cells/min (*n* = 7); *α*-syn: 549.8 ± 93.88 pmol/mio cells/min (*n* = 7); two-tailed paired *t* test: *t* = 3.838, df = 6, ∗∗*P* = .0086. (F) K_M_ for 5-HT at SERT: ctrl: K_M_ = 2.0 ± 0.19 *μ*M; *α*-syn: K_M_ = 2.5 ± 0.27 *μ*M; two-tailed paired *t* test: *t* = 2.038, df = 6, *P* = .0877. (Immunoblots show expression levels of DAT (G) or SERT (H) in control (left lane) and after *α*-syn overexpression (right lane). Quantification of DAT (G, right panel) (control: 0.9 ± 0.19; *n* = 6; *α*-syn: 1.4 ± 0.15; *n* = 6; two-tailed paired *t* test: *t* = 2.704, df = 5, ∗*P* = .0426). Quantification of SERT (H, right panel) (control: 1.4 ± 0.16; *n* = 5; *α*-syn: 1.6 ± 0.14; *n* = 5; two-tailed paired *t* test: *t* = 2.972, df = 4, ∗*P* = .0411); bands were normalized to GAPDH.

To determine whether the increased uptake was contingent on changes in transporter protein expression, we prepared total cell lysates and performed immunoblotting. Western analysis revealed increased total protein expression of DAT and SERT in the presence of *α*-syn (Fig. 3, G and H; Supplemental Figs. 3 and 4), corresponding to the observed increase in substrate uptake. Note, that the diffuse banding pattern observed for DAT and SERT reflects heterogeneous glycosylation, which results in multiple species with distinct apparent molecular weights (between 70 and 100 kD) on the blot.

### 3.4 Knockdown of *α*-syn in HEK-293 cells reduces DAT and SERT uptake and expression

To assess the role of endogenous *α*-syn in monoamine transporter regulation, HEK-293 cells were transfected with siRNAs targeting *SNCA* or control siRNA. Twenty-four hours later, cells were transfected with DAT or SERT expression plasmids, and uptake assays were performed the following day.

Note, that uptake experiments after siRNA-mediated knockdown were performed at a single substrate concentration below the K_M_ to sensitively detect changes in transporter capacity because cell numbers limited experimental throughput and full kinetic analysis.

siRNA-mediated knockdown of *α*-syn significantly reduced uptake at both DAT (Fig. 4A) and SERT (Fig. 4B). Immunoblot analysis confirmed efficient reduction of *α*-syn protein levels (Fig. 4, C and D; Supplemental Figs. 5 and 6). In parallel, DAT and SERT protein expression levels were reduced under *α*-syn knockdown conditions (Fig. 4, C and D; Supplemental Figs. 5 and 6).

**Fig. 4:**
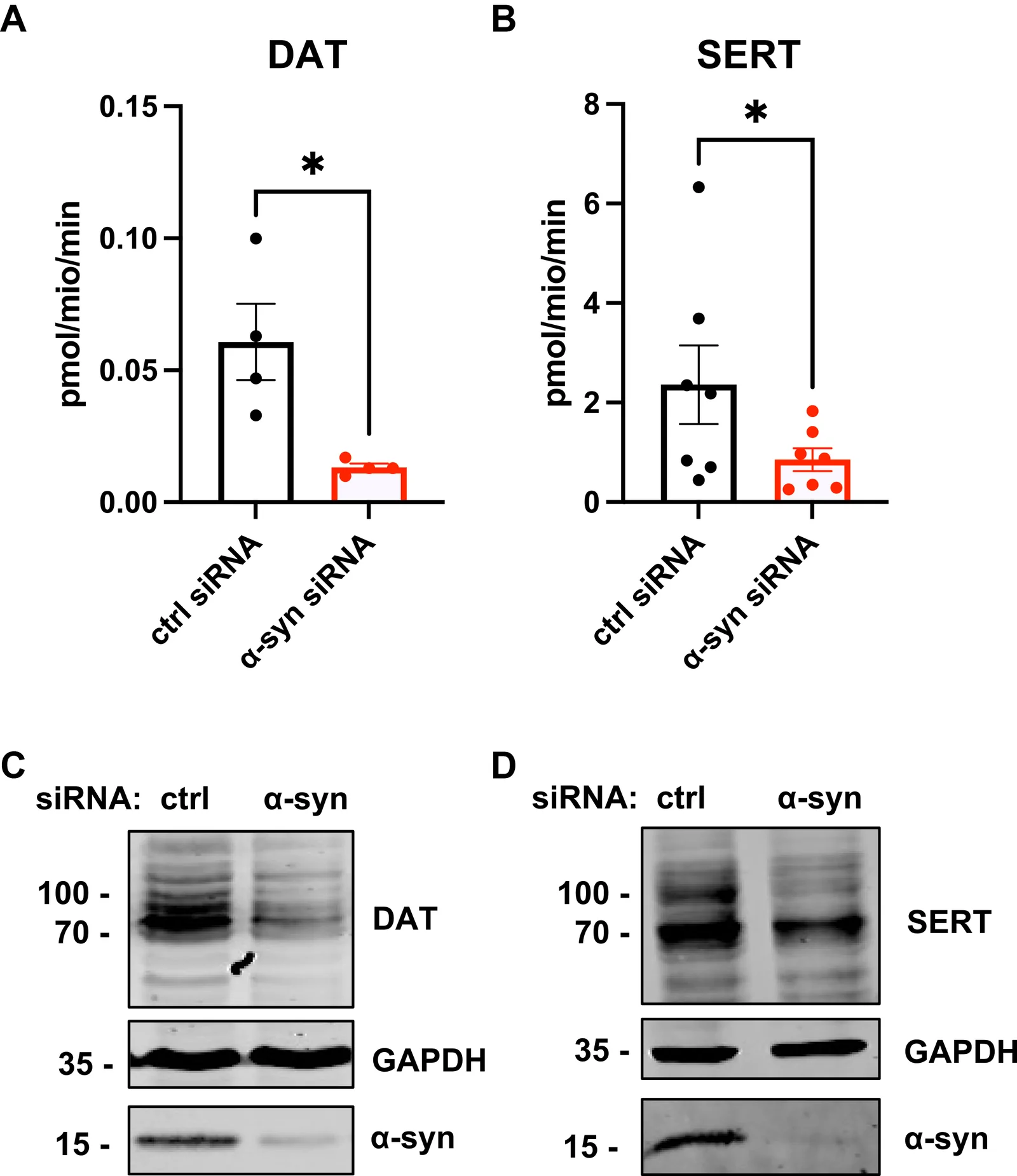
Knockdown of *α*-syn reduces monoamine uptake and transporter expression. (A) DA uptake in DAT expressing HEK-293 cells after expression of control (ctrl) or *α*-syn siRNAs: ctrl: 0.06 ± 0.014 pmol/mio cells/min (*n* = 4); *α*-syn: 0.01 ± 0.001 pmol/mio cells/min (*n* = 4); two-tailed paired *t* test: *t* = 3.398, df = 3, ∗*P* = .0425. (B) 5-HT uptake in SERT expressing HEK-293 cells after expression of control (ctrl) or *α*-syn siRNAs: ctrl: 2.3 ± 0.79 pmol/mio cells/min (*n* = 7); *α*-syn: 0.8 ± 0.23 pmol/mio cells/min (*n* = 7); two-tailed paired *t* test: *t* = 2.536, df = 6, ∗*P* = .0443. Immunoblots for DAT (C) and SERT (D), GAPDH and *α*-syn.

### 3.5 Knockout of *α*-syn in HEK-293 cells reduces DAT and SERT uptake and expression

To further examine the contribution of endogenous *α*-syn, we generated an *α*-syn knockout HEK-293 cell line using CRISPR/Cas9-mediated gene editing. Successful deletion of *SNCA* was confirmed by immunoblotting (Fig. 5, G and H; Supplemental Fig. 7).

**Fig. 5:**
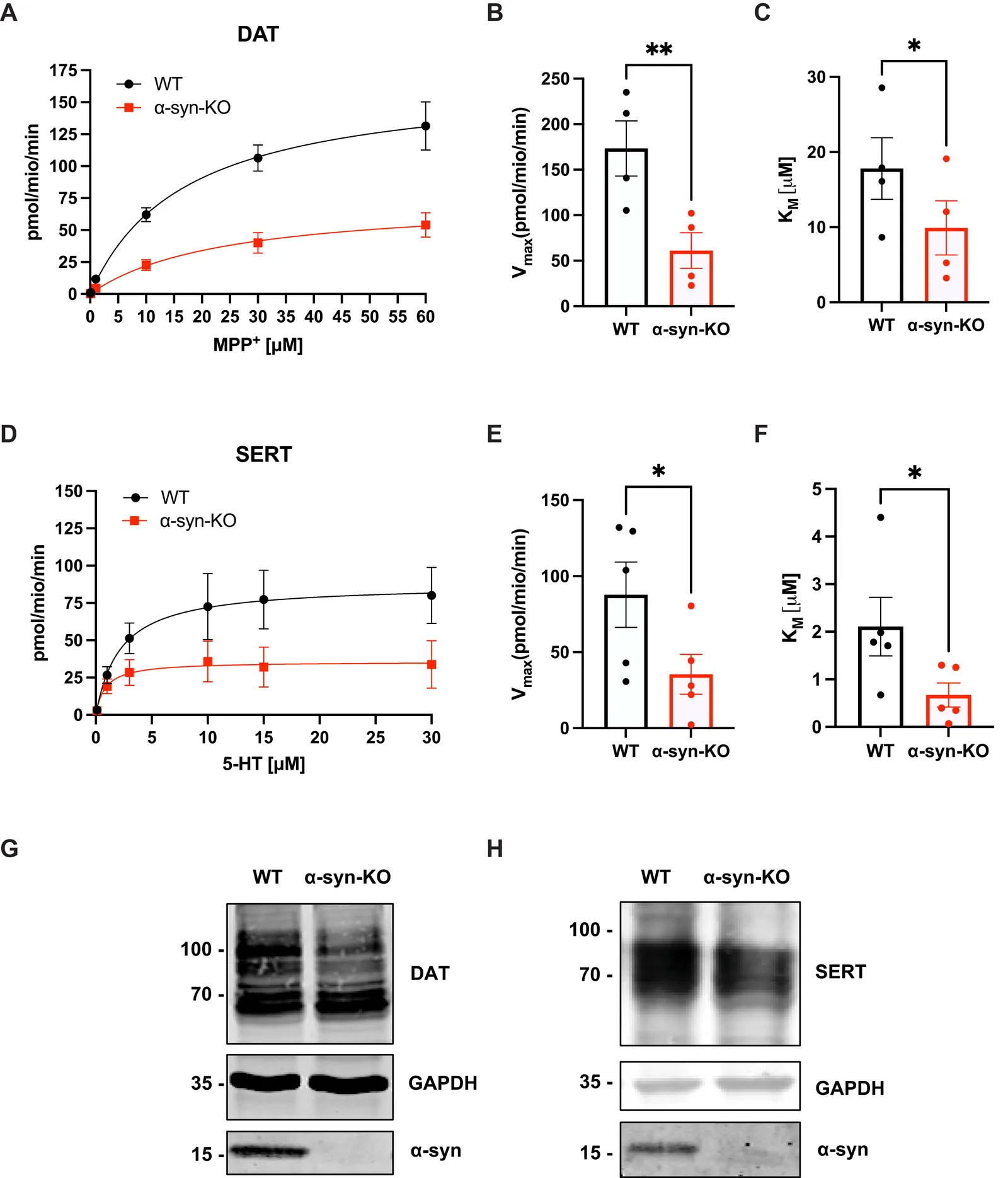
Knockout of *α*-syn reduces V_max_ and K_M_ at DAT and SERT. (A) Saturation uptake of MPP^+^ in wild-type (WT) of *α*-syn-knockout (KO) cells transfected with human DAT. (B) V_max_ of MPP^+^ uptake at DAT: V_max_ for MPP^+^ uptake at DAT: ctrl V_max_: 173.4 ± 30.26 pmol/mio cells/min (*n* = 4); *α*-syn: 61.1 ± 19.52 pmol/mio cells/min (*n* = 4); two-tailed paired *t* test: *t* = 7.122, df = 3, ∗∗*P* = .0057. (C) K_M_ of MPP^+^ at DAT: ctrl: K_M_ = 17.8 ± 4.10 *μ*M (*n* = 4); *α*-syn: K_M_ = 9.92 ± 3.59 *μ*M (*n* = 4); two-tailed paired *t* test: *t* = 3.812, df = 3, ∗*P* = .0317. (D) Saturation uptake of 5-HT in wild-type of *α*-syn-KO cells transfected with human SERT. (E) V_max_ for 5-HT uptake at SERT: ctrl V_max_: 87.8 ± 21.50 pmol/mio cells/min (*n* = 5); *α*-syn: 35.4 ± 13.11 pmol/mio cells/min (*n* = 5); two-tailed paired *t* test: *t* = 3.662, df = 4, ∗*P* = .0215. (F) K_M_ of 5-HT at SERT: ctrl: K_M_ = 2.1 ± 0.61 *μ*M (*n* = 5); *α*-syn: K_M_ = 0.7 ± 0.25 *μ*M (n =5); two-tailed paired *t* test: *t* = 2.785, df = 4, ∗*P* = .0496. Immunoblot showing expression levels of DAT (G), SERT (H), GAPDH and *α*-syn.

*α*-Syn knockout and wild-type HEK-293 cells were transfected with DAT or SERT, and uptake kinetics were assessed. Loss of *α*-syn resulted in significantly reduced V_max_ values for both DAT ([^3^H]-MPP^+^) and SERT ([^3^H]-5-HT) (Fig. 5, A, B, D, and E). In contrast to overexpression and knockdown conditions, K_M_ values were significantly decreased in *α*-syn knockout cells (Fig. 5, C and F).

Consistent with reduced uptake, total DAT and SERT protein expression levels were also decreased in *α*-syn knockout cells (Fig. 5, G and H; Supplemental Figs. 7 and 8).

### 3.6 Knockout of *α*-syn attenuates amphetamine-triggered reverse transport at DAT and SERT

Monoamine transporters can operate in reverse, facilitating substrate efflux in response to psychostimulants such as amphetamines.^20^ To assess whether *α*-syn influences reverse transport at DAT or SERT, we measured amphetamine-induced efflux after *α*-syn overexpression or in *α*-syn knockout cells. We decided to use 10 *μ*M D-amphetamine at DAT and 10 *μ*M MDMA (“ecstasy”) at SERT to trigger efflux as this is roughly 10-fold above the EC_50_ reported for amphetamine-induced efflux at these transporters.^21^^,^^22^

Although *α*-syn overexpression did not significantly alter amphetamine-triggered DAT mediated [^3^H]-MPP^+^ efflux (Fig. 6, A and B), MDMA-triggered [^3^H]-5-HT efflux at SERT was significantly elevated (Fig. 6, E and F**)**. In contrast, *α*-syn knockout cells displayed a marked reduction in efflux of [^3^H]-MPP^+^ at DAT and [^3^H]-5-HT at SERT compared with wild-type controls (Fig. 6, C, D, G, and H), indicating that endogenous *α*-syn is required for normal reverse transport. Interestingly, baseline efflux was also significantly elevated in SERT- but not DAT-transfected *α*-syn knockout cells.

**Fig. 6:**
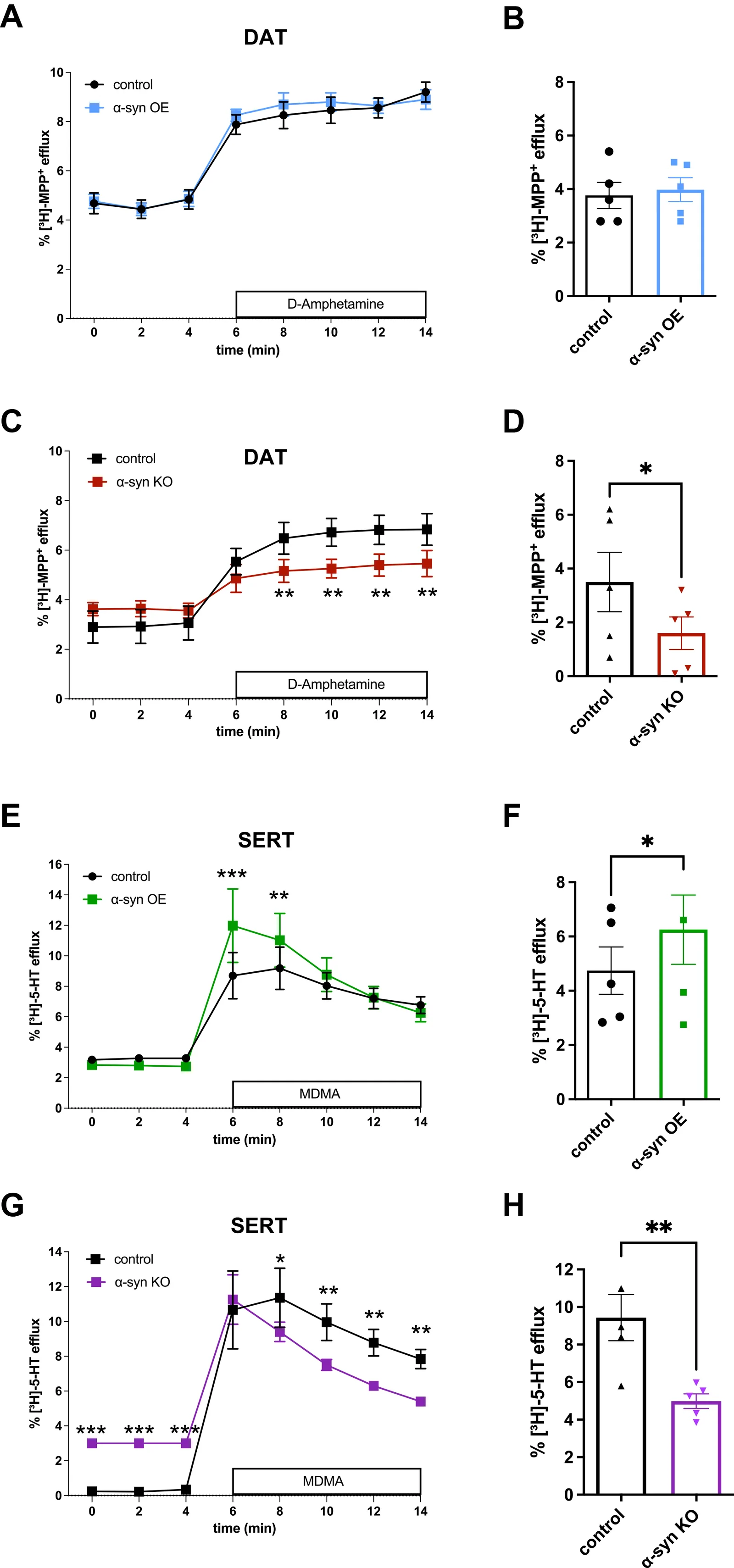
Effects of *α*-syn on amphetamine-triggered efflux at DAT and SERT. HEK-293 cells overexpressing (OE) *α*-syn or control (pcDNA3.1) and DAT (A, B) or SERT (E, F) were superfused with buffer, and D-amphetamine (10 *μ*M; DAT) or MDMA (10 *μ*M; SERT) were added at minute 6 to induce MPP^+^ (DAT) or 5-HT (SERT) efflux. (A) DAT and *α*-syn OE: two-way repeated measures ANOVA followed by Sidak’s multiple comparisons test: time: *F*(7,28) = 49.7, ∗∗∗*P* < .0001; no significant treatment or time × treatment effect. (B) Percent of MPP^+^ efflux after OE (control: 3.8% ± 0.49%; *n* = 5; *α*-syn: 3.9% ± 0.45%; *n* = 5; two-tailed paired *t* test: *t* = 0.6116, df = 4, *P* = .5739) Superfusion of *α*-syn-knockout (KO) or wild-type (WT) cells transfected with DAT (C, D) or SERT (G, H). (C) DAT and *α*-syn-KO: two-way repeated measures ANOVA followed by Sidak’s multiple comparisons test: time: *F*(7,28) = 6.7, ∗∗∗*P* = .0001; treatment: *F*(1,4) = 14.7, ∗*P* = .0184, time × treatment: *F*(7,28) = 8.3, ∗∗∗*P* < .0001. (D) Percent of MPP^+^ efflux in *α*-syn-KO cells (control: 3.5% ± 1.11%; *n* = 5; *α*-syn-KO: 1.6% ± 0.60%; *n* = 5; two-tailed paired *t* test: *t* = 2.946, df = 4, ∗*P* = .0421). (E) SERT and *α*-syn OE: two-way repeated measures ANOVA followed by Sidak’s multiple comparisons test: time: *F*(7,28) = 18.09, ∗∗∗*P* < .0001; treatment: *F*(1,4) = 3.413, *P* = .1384, time × treatment: *F*(7,28) = 10.05, ∗∗∗*P* < .0001. (F) Percent of 5-HT efflux in *α*-syn-OE cells (control: 4.7% ± 0.87%; *n* = 5; *α*-syn-KO: 6.3% ± 1.28%; *n* = 5; two-tailed paired *t* test: *t* = 3.036, df = 4, ∗*P* = .0385) (G) SERT and *α*-syn: two-way repeated measures ANOVA followed by Sidak’s multiple comparisons test: time: *F*(7,28) = 37.46, ∗∗∗*P* < .0001; treatment: *F*(1,4) = 0.02062, *P* = .8928, time × treatment: *F*(7,28) = 18.39, ∗∗∗*P* < .0001. (H) Percent of 5-HT efflux in *α*-syn-KO cells (control: 9.4% ± 1.23%; *n* = 5; *α*-syn-KO: 5.0% ± 0.39%; *n* = 5; two-tailed paired *t* test: *t* = 4.975, df = 4, ∗∗*P* = .0076).

## 4 Discussion

*α*-Syn remains an enigmatic protein despite decades of intensive research. Although *α*-syn is clearly linked to PD and other synucleinopathies, the molecular mechanisms by which it triggers neurodegeneration remain incompletely understood.^23^ This lack of a clear pathogenic mechanism may in part reflect our limited understanding of the physiological roles of *α*-syn.^3^ In this context, it is notable that recent clinical trials targeting *α*-syn with immunotherapies failed to demonstrate clear disease modification in patients with PD,^24^^,^^25^ highlighting the need for a more refined understanding of *α*-syn biology.

Here, we investigated how *α*-syn modulates the function of the monoamine transporters DAT and SERT. Both transporters belong to the SLC6 family of neurotransmitter:sodium symporters and use the sodium gradient to mediate uptake of monoamines from the extracellular space back into presynaptic neurons, thereby playing a central role in monoamine homeostasis and as targets for psychostimulants, antidepressants and neurotoxicants.^26^^,^^27^ Within the central nervous system, DAT and SERT are selectively expressed in dopaminergic and serotonergic neurons, respectively. Notably, both neuronal populations are particularly susceptible to degeneration in PD,^28^ although the mechanisms underlying this selective vulnerability remain unclear.

Previous studies have shown that *α*-syn can directly interact with DAT,^7^^,^^10^ SERT,^9^ and the closely related NET,^8^ suggesting a potential role for *α*-syn in regulating monoamine transporter function. However, the functional consequences of these interactions have remained controversial, as both increases and decreases in transporter activity have been reported after *α*-syn overexpression in different cellular and in vivo models.^7^, ^8^, ^9^, ^10^, ^11^, ^12^, ^13^, ^14^ To address these inconsistencies, we systematically manipulated *α*-syn expression levels using overexpression, knockdown, and knockout approaches in well known cellular systems.

Our results demonstrate that increased *α*-syn expression enhanced DAT- and SERT-mediated uptake, primarily through an increase in transport capacity (V_max_), without affecting substrate affinity. This increase in uptake was accompanied by elevated total transporter protein levels, suggesting that *α*-syn positively regulates DAT and SERT expression. Conversely, reducing *α*-syn levels via siRNA-mediated knockdown or complete genetic deletion resulted in decreased transporter expression and reduced uptake. Although we measured total DAT and SERT protein, functional uptake scales with the surface-expressed transporter fraction.^16^ Therefore, the observed *α*-syn-dependent changes in uptake likely reflect altered functional transporter density at the plasma membrane.

Together, these findings indicate that *α*-syn acts as a positive regulator of DAT and SERT expression and function, although the precise molecular mechanisms remain to be elucidated.

Several nonmutually exclusive mechanisms could explain how *α*-syn modulates transporter expression. *α*-Syn has been implicated in vesicular trafficking and membrane dynamics,^29^ raising the possibility that it affects secretory trafficking or endocytic recycling of monoamine transporters. Alternatively, *α*-syn may influence transporter stability or turnover, or exert indirect effects on transporter gene expression.^30^ Dissecting these possibilities will require future studies addressing transporter surface expression, trafficking kinetics, and transcriptional regulation.

Our finding that *α*-syn positively modulates DAT expression is supported by recent human imaging data: *SNCA* variants associated with increased *α*-syn expression identified in de novo patients with PD were linked to elevated striatal DAT uptake.^31^ It is well established that duplications and triplications of *SNCA* cause increased *α*-syn expression and early-onset familial PD.^32^, ^33^, ^34^, ^35^ It is therefore conceivable that elevated *α*-syn levels may directly increase DAT (and potentially SERT) expression in human neurons with direct effects on physiology and pathology. Interestingly, we recently observed that the cell-type specific overexpression of *α*-syn in mouse DA neurons resulted in increased striatal DA tissue levels,^15^ an effect that is consistent with a positive modulation of DAT function and/or expression by *α*-syn. These findings are also in line with findings from SNCA^−/−^ mice, which displayed reduced striatal DA levels suggesting that decreased DA storage and/or decreased DA synthesis resulted from loss of *α*-syn.^6^ Of course, these effects may not be solely attributable to *α*-syn-mediated regulation of DAT but could also reflect altered vesicular DA sequestration through vesicular monoamine transporter 2, another presynaptic protein known to interact with *α*-syn.^36^ Knockdown of *α*-syn for instance increased the density of vesicular monoamine transporter 2 in differentiated SH-SY5Y cells,^37^ whereas overexpression of wild-type *α*-syn^36^ or *α*-syn^A53T^^38^ reduced uptake via vesicular monoamine transporter 2 in SH-SY5Y cells.

Increased DAT expression has been associated with enhanced oxidative stress and dopaminergic neurodegeneration.^39^^,^^40^ Thus, *α*-syn-induced increases in DAT expression may contribute to the heightened vulnerability of dopaminergic neurons in PD and may be related to the earlier onset of disease in SNCA duplication or triplication carriers.^33^^,^^34^ The idea that higher DAT levels put neurons at risk is further supported by regional differences in DAT expression within the midbrain: dopaminergic neurons in the ventral tegmental area and dorsolateral substantia nigra *pars compacta* express lower levels of DAT and are relatively resistant to degeneration in PD and in toxin-based animal models.^41^, ^42^, ^43^ The current findings therefore raise the possibility that *α*-syn–dependent regulation of DAT (and SERT) expression contributes to selective neuronal vulnerability.

Interestingly, although *α*-syn overexpression or knockdown did not alter substrate affinity at DAT or SERT, complete loss of *α*-syn resulted in decreased K_M_ values for both transporters, suggesting increased apparent substrate affinity. This observation raises the possibility that *α*-syn not only regulates transporter abundance but may also modulate transporter conformation and/or function directly. One intriguing hypothesis is that *α*-syn forms part of a functional complex with monoamine transporters at the plasma membrane, thereby influencing transport kinetics under physiological conditions. This is also supported by previous work showing a direct interaction between DAT and *α*-syn.^7^

Of note, one limitation of the present study is that the CRISPR/Cas9 experiments were conducted using a single SNCA^−/−^ HEK-293 clone. Consequently, potential clone-specific effects, including off-target Cas9 editing, epigenetic alterations, or compensatory transcriptional adaptations, cannot be completely excluded.

In addition to mediating monoamine reuptake, monoamine transporters can operate in reverse to promote substrate efflux, particularly in response to psychostimulants such as amphetamines.^20^ Amphetamine-induced reverse transport is primarily driven by changes in ionic gradients but is also regulated by intracellular signaling pathways, including protein kinase C^44^ and Ca^2+^/calmodulin-dependent protein kinase II.^45^, ^46^, ^47^ A previous study showed that overexpression of eCFP-*α*-syn elevated amphetamine-triggered DA efflux in CHO cells.^48^ Although *α*-syn overexpression did not directly affect amphetamine-induced transporter-mediated efflux at DAT, we observed that MDMA caused significantly more 5-HT efflux in SERT expressing cells. Whether this finding reflects a genuine transporter-specific effect of *α*-syn overexpression or simply technical variability remains to be determined.

In contrast, knockout of *α*-syn significantly attenuated substrate efflux at both, DAT and SERT. Although cells were preloaded with [^3^H]-labeled substrates under conditions designed to promote intracellular accumulation, both substrate loading and subsequent efflux remain dependent on transporter expression levels. Accordingly, the reduced efflux observed in *α*-syn-deficient cells is most parsimoniously explained by decreased transporter surface expression and transport capacity, rather than a change in intrinsic efflux properties per transporter. Finally, basal efflux at SERT appeared to be increased in *α*-syn-deficient cells; however, it remains unclear whether this reflects enhanced baseline efflux or is instead a consequence of reduced substrate uptake used for normalization, rather than a true increase in constitutive substrate release.

What might explain the conflicting reports in the literature regarding *α*-syn-dependent regulation of monoamine transporters?

Discrepancies likely arise from differences in experimental models, expression levels, and cellular context. Although heterologous cell systems offer powerful reductionist approaches, they lack many of the regulatory components present in native neurons. Additional complexity arises from the existence of multiple *α*-syn molecular species, such as monomers, oligomers, and higher-order species including aggregates, as well as diverse post-translational modifications such as phosphorylation or acetylation, all of which may differentially affect *α*-syn function and pathogenicity.^49^, ^50^, ^51^ Our study focused on wild-type *α*-syn monomers expressed in cellular systems; we did not assess oligomeric or post-translationally modified species, which may differentially modulate transporter function and could contribute to variability in prior reports.

In summary, our study provides further evidence that *α*-syn is an important regulator of monoamine transporter expression and function. By modulating DAT and SERT levels, *α*-syn may influence monoamine homeostasis and contribute to selective neuronal vulnerability in PD. A deeper understanding of the molecular mechanisms underlying this regulation may provide new insight into both the physiological roles of *α*-syn and its contribution to neurodegenerative disease.

## Conflict of interest

The authors declare no conflicts of interest.

## References

[1] Spillantini M.G., Schmidt M.L., Lee V.M., Trojanowski J.Q., Jakes R., Goedert M. (1997). Alpha-synuclein in Lewy bodies. Nature.

[2] Polymeropoulos M.H., Lavedan C., Leroy E. (1997). Mutation in the alpha-synuclein gene identified in families with Parkinson’s disease. Science.

[3] Burré J., Edwards R.H., Halliday G. (2024). Research priorities on the role of *α*-synuclein in Parkinson’s disease pathogenesis. Mov Disord.

[4] Bendor J.T., Logan T.P., Edwards R.H. (2013). The function of *α*-synuclein. Neuron.

[5] Sulzer D., Edwards R.H. (2019). The physiological role of *α*-synuclein and its relationship to Parkinson’s disease. J Neurochem.

[6] Abeliovich A., Schmitz Y., Fariñas I. (2000). Mice lacking alpha-synuclein display functional deficits in the nigrostriatal dopamine system. Neuron.

[7] Lee F.J., Liu F., Pristupa Z.B., Niznik H.B. (2001). Direct binding and functional coupling of alpha-synuclein to the dopamine transporters accelerate dopamine-induced apoptosis. FASEB J.

[8] Wersinger C., Jeannotte A., Sidhu A. (2006). Attenuation of the norepinephrine transporter activity and trafficking via interactions with alpha-synuclein. Eur J Neurosci.

[9] Wersinger C., Rusnak M., Sidhu A. (2006). Modulation of the trafficking of the human serotonin transporter by human alpha-synuclein. Eur J Neurosci.

[10] Wersinger C., Sidhu A. (2003). Attenuation of dopamine transporter activity by alpha-synuclein. Neurosci Lett.

[11] Chadchankar H., Ihalainen J., Tanila H., Yavich L. (2011). Decreased reuptake of dopamine in the dorsal striatum in the absence of *α*-synuclein. Brain Res.

[12] Lundblad M., Decressac M., Mattsson B., Björklund A. (2012). Impaired neurotransmission caused by overexpression of *α*-synuclein in nigral dopamine neurons. Proc Natl Acad Sci U S A.

[13] Swant J., Goodwin J.S., North A. (2011). *α*-Synuclein stimulates a dopamine transporter-dependent chloride current and modulates the activity of the transporter. J Biol Chem.

[14] Threlfell S., Mohammadi A.S., Ryan B.J. (2021). Striatal dopamine transporter function is facilitated by converging biology of *α*-synuclein and cholesterol. Front Cell Neurosci.

[15] Garcia Moreno S.I., Limani F., Ludwig I. (2024). Viral overexpression of human alpha-synuclein in mouse substantia nigra dopamine neurons results in hyperdopaminergia but no neurodegeneration. Exp Neurol.

[16] Sucic S., Bönisch H., Bönisch H., Sitte H.H. (2016). Neurotransmitter Transporters: Investigative Methods.

[17] Steinkellner T., Mayer F.P., Hofmaier T., Bönisch H., Sitte H.H. (2016). Neurotransmitter Transporters: Investigative Methods.

[18] Lee B.R., Kamitani T. (2011). Improved immunodetection of endogenous *α*-synuclein. PLoS One.

[19] Qi Y., Wang J.K., McMillian, Chikaraishi D.M. (1997). Characterization of a CNS cell line, CAD, in which morphological differentiation is initiated by serum deprivation. J Neurosci.

[20] Sitte H.H., Freissmuth M. (2015). Amphetamines, new psychoactive drugs and the monoamine transporter cycle. Trends Pharmacol Sci.

[21] Pifl C., Drobny H., Reither H., Hornykiewicz O., Singer E.A. (1995). Mechanism of the dopamine-releasing actions of amphetamine and cocaine: plasmalemmal dopamine transporter versus vesicular monoamine transporter. Mol Pharmacol.

[22] Hilber B., Scholze P., Dorostkar M.M. (2005). Serotonin-transporter mediated efflux: a pharmacological analysis of amphetamines and non-amphetamines. Neuropharmacology.

[23] Calabresi P., Di Lazzaro G., Marino G., Campanelli F., Ghiglieri V. (2023). Advances in understanding the function of alpha-synuclein: implications for Parkinson’s disease. Brain.

[24] Pagano G., Taylor K.I., Anzures-Cabrera J. (2022). Trial of prasinezumab in early-stage Parkinson’s disease. N Engl J Med.

[25] Lang A.E., Siderowf A.D., Macklin E.A. (2022). Trial of cinpanemab in early Parkinson’s disease. N Engl J Med.

[26] Kristensen A.S., Andersen J., Jørgensen T.N. (2011). SLC6 neurotransmitter transporters: structure, function, and regulation. Pharmacol Rev.

[27] Steinkellner T., Freissmuth M., Sitte H.H., Montgomery T. (2011). The ugly side of amphetamines: short- and long-term toxicity of 3,4-methylenedioxymethamphetamine (MDMA, “Ecstasy”), methamphetamine and D-amphetamine. Biol Chem.

[28] Giguère N., Burke Nanni S., Trudeau L.E. (2018). On cell loss and selective vulnerability of neuronal populations in Parkinson’s disease. Front Neurol.

[29] Lautenschläger J., Kaminski C.F., Kaminski Schierle G.S. (2017). *α*-Synuclein – regulator of exocytosis, endocytosis, or both?. Trends Cell Biol.

[30] Somayaji M., Lanseur Z., Choi S.J., Sulzer D., Mosharov E.V. (2021). Roles for *α*-synuclein in gene expression. Genes (Basel).

[31] Alhusaini S., Dayanim G., Kandil M. (2025). Parkinson disease SNCA risk variants are associated with higher asymmetric putamen dopaminergic dysfunction. Neurol Genet.

[32] Byers B., Cord B., Nguyen H.N. (2011). SNCA triplication Parkinson’s patient’s iPSC-derived DA neurons accumulate *α*-synuclein and are susceptible to oxidative stress. PLoS One.

[33] Chartier-Harlin M.C., Kachergus J., Roumier C. (2004). Alpha-synuclein locus duplication as a cause of familial Parkinson’s disease. Lancet.

[34] Singleton A.B., Farrer M., Johnson J. (2003). Alpha-synuclein locus triplication causes Parkinson’s disease. Science.

[35] Wurster I., Quadalti C., Rossi M. (2022). Linking the phenotype of SNCA triplication with PET-MRI imaging pattern and alpha-synuclein CSF seeding. NPJ Parkinsons Dis.

[36] Guo J.T., Chen A.Q., Kong Q., Zhu H., Ma C.M., Qin C. (2008). Inhibition of vesicular monoamine transporter-2 activity in alpha-synuclein stably transfected SH-SY5Y cells. Cell Mol Neurobiol.

[37] Fountaine T.M., Venda L.L., Warrick N. (2008). The effect of alpha-synuclein knockdown on MPP+ toxicity in models of human neurons. Eur J Neurosci.

[38] Lotharius J., Barg S., Wiekop P., Lundberg C., Raymon H.K., Brundin P. (2002). Effect of mutant alpha-synuclein on dopamine homeostasis in a new human mesencephalic cell line. J Biol Chem.

[39] Chen L., Ding Y., Cagniard B. (2008). Unregulated cytosolic dopamine causes neurodegeneration associated with oxidative stress in mice. J Neurosci.

[40] Masoud S.T., Vecchio L.M., Bergeron Y. (2015). Increased expression of the dopamine transporter leads to loss of dopamine neurons, oxidative stress and l-DOPA reversible motor deficits. Neurobiol Dis.

[41] Fearnley J.M., Lees A.J. (1991). Ageing and Parkinson’s disease: substantia nigra regional selectivity. Brain.

[42] Afonso-Oramas D., Cruz-Muros I., Alvarez de la Rosa D. (2009). Dopamine transporter glycosylation correlates with the vulnerability of midbrain dopaminergic cells in Parkinson’s disease. Neurobiol Dis.

[43] Dauer W., Przedborski S. (2003). Parkinson’s disease: mechanisms and models. Neuron.

[44] Johnson L.A., Guptaroy B., Lund D., Shamban S., Gnegy M.E. (2005). Regulation of amphetamine-stimulated dopamine efflux by protein kinase C beta. J Biol Chem.

[45] Steinkellner T., Montgomery T.R., Hofmaier T. (2015). Amphetamine action at the cocaine- and antidepressant-sensitive serotonin transporter is modulated by *α*CaMKII. J Neurosci.

[46] Steinkellner T., Yang J.W., Montgomery T.R. (2012). Ca(2+)/calmodulin-dependent protein kinase II*α* (*α*CaMKII) controls the activity of the dopamine transporter: implications for Angelman syndrome. J Biol Chem.

[47] Fog J.U., Khoshbouei H., Holy M. (2006). Calmodulin kinase II interacts with the dopamine transporter C terminus to regulate amphetamine-induced reverse transport. Neuron.

[48] Butler B., Saha K., Rana T. (2015). Dopamine transporter activity is modulated by *α*-synuclein. J Biol Chem.

[49] Zhang S., Zhu R., Pan B. (2023). Post-translational modifications of soluble *α*-synuclein regulate the amplification of pathological *α*-synuclein. Nat Neurosci.

[50] Parra-Rivas L.A., Madhivanan K., Aulston B.D. (2023). Serine-129 phosphorylation of *α*-synuclein is an activity-dependent trigger for physiologic protein-protein interactions and synaptic function. Neuron.

[51] Ramalingam N., Jin S.X., Moors T.E. (2023). Dynamic physiological *α*-synuclein S129 phosphorylation is driven by neuronal activity. NPJ Parkinsons Dis.

