# Supplementary material for: α-Synuclein regulates monoamine transporter capacity and amphetamine-induced efflux via modulation of transporter expression
Source: J Pharmacol Exp Ther. 2026 Jul 4;393(8):104981. doi: 10.1016/j.jpet.2026.104981 (PMC13688989; doi:10.1016/j.jpet.2026.104981)
Supplement: Supplementary file 1 [file mmc1.pdf]

Alpha-Synuclein Regulates Monoamine Transporter Capacity and Amphetamine-Induced Efflux via Modulation of Transporter Expression

Iina Ludwig[1], Samuel Moser[1], Marina Lukenic[1], Michael Connor[1], Amila Sofic [1], Samantha Kaltenbrunner[1], Constantin Pätzold[1], Alexander Hausleitner[1], Catherine Gilbert[1], Sonja Sucic[1], Marko Roblek[1], Thomas Steinkellner[1,*]

[1] Institute of Pharmacology, Center for Physiology and Pharmacology, Medical University of Vienna, Austria

*** Corresponding Author:**

Thomas Steinkellner, PhD

Institute of Pharmacology

Center for Physiology and Pharmacology

Medical University of Vienna

Waehringerstr. 13a, 1090 Vienna, Austria

Phone: 0043-1-40160-313-76

Email: thomas.steinkellner@meduniwien.ac.at

Supplemental Figure 1:

Immunoblots for N2a cells overexpressing different ratios of hSERT and human α-syn.

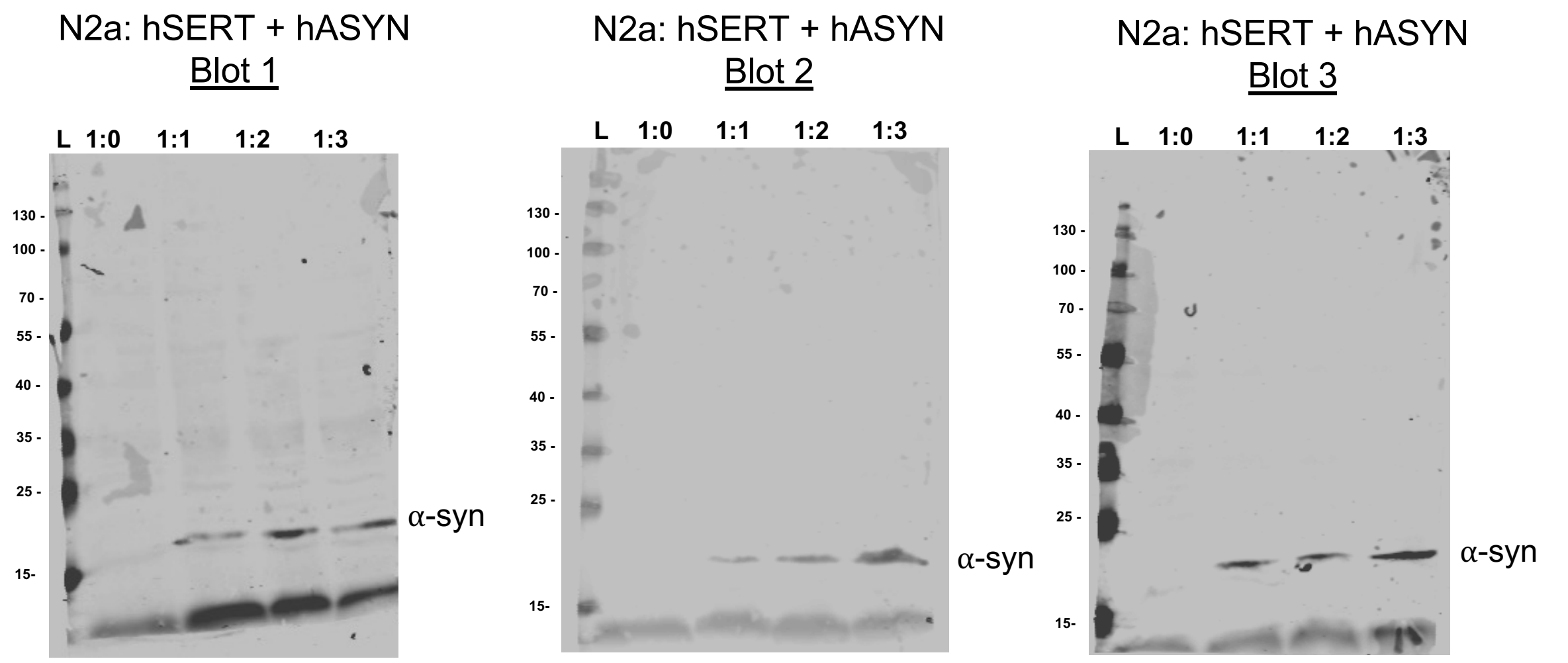

WB: 15G7 (human α-synuclein)

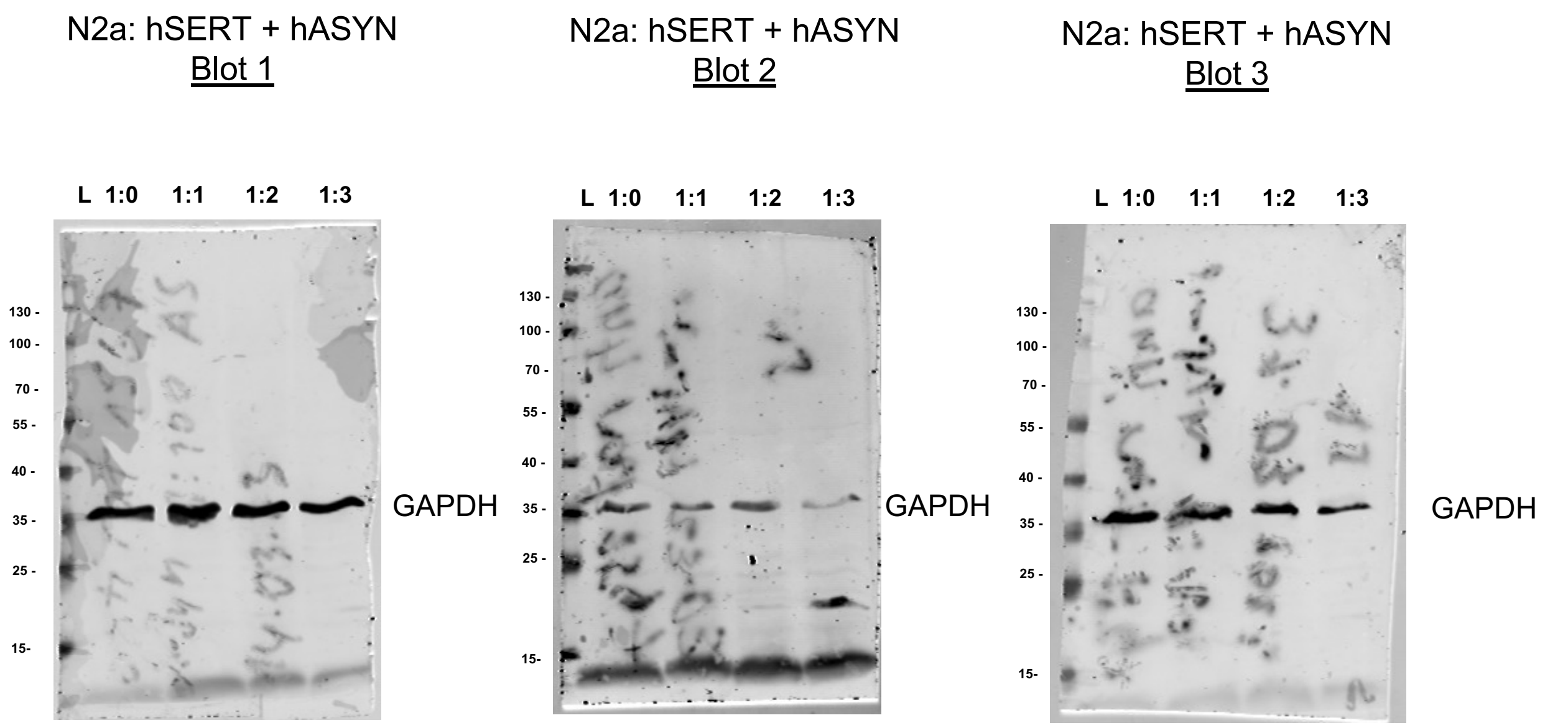

WB: GAPDH

Supplemental Figure 2:

Overexpression of α-syn in HEK-293 cells increases DAT-mediated [^{3}H]MPP$^+$ uptake.

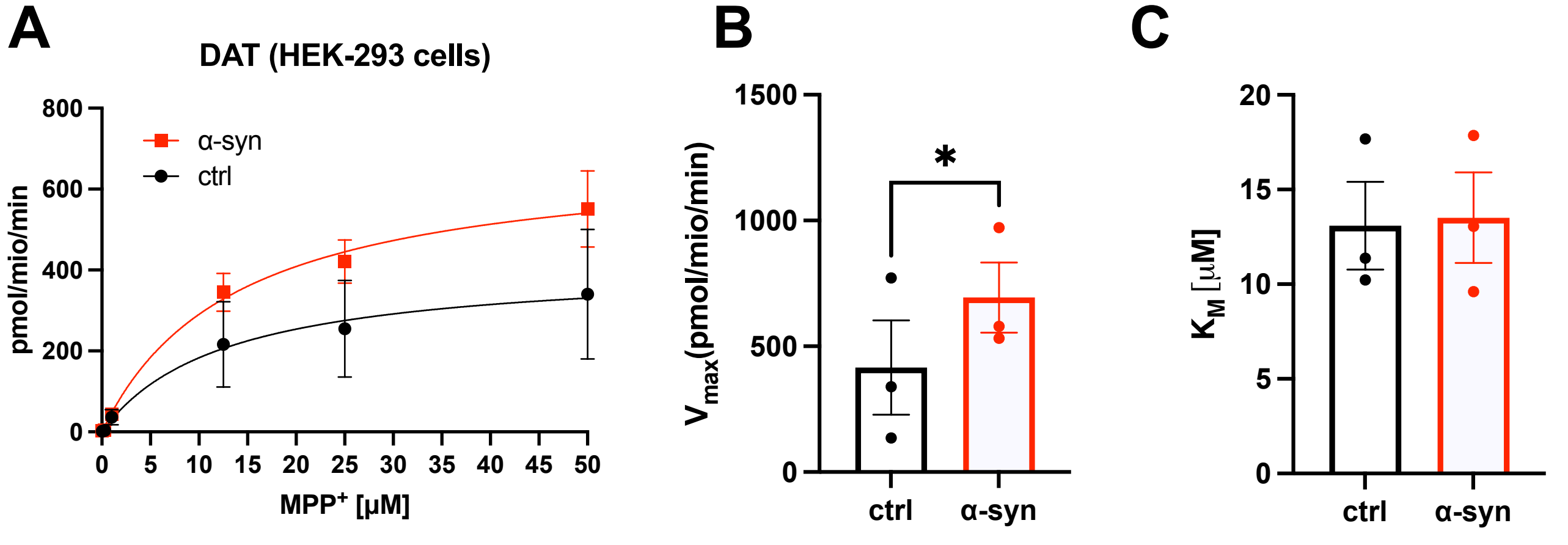

Supplemental Figure 3:

Immunoblots for HEK-293 cells overexpressing α-syn and hDAT.

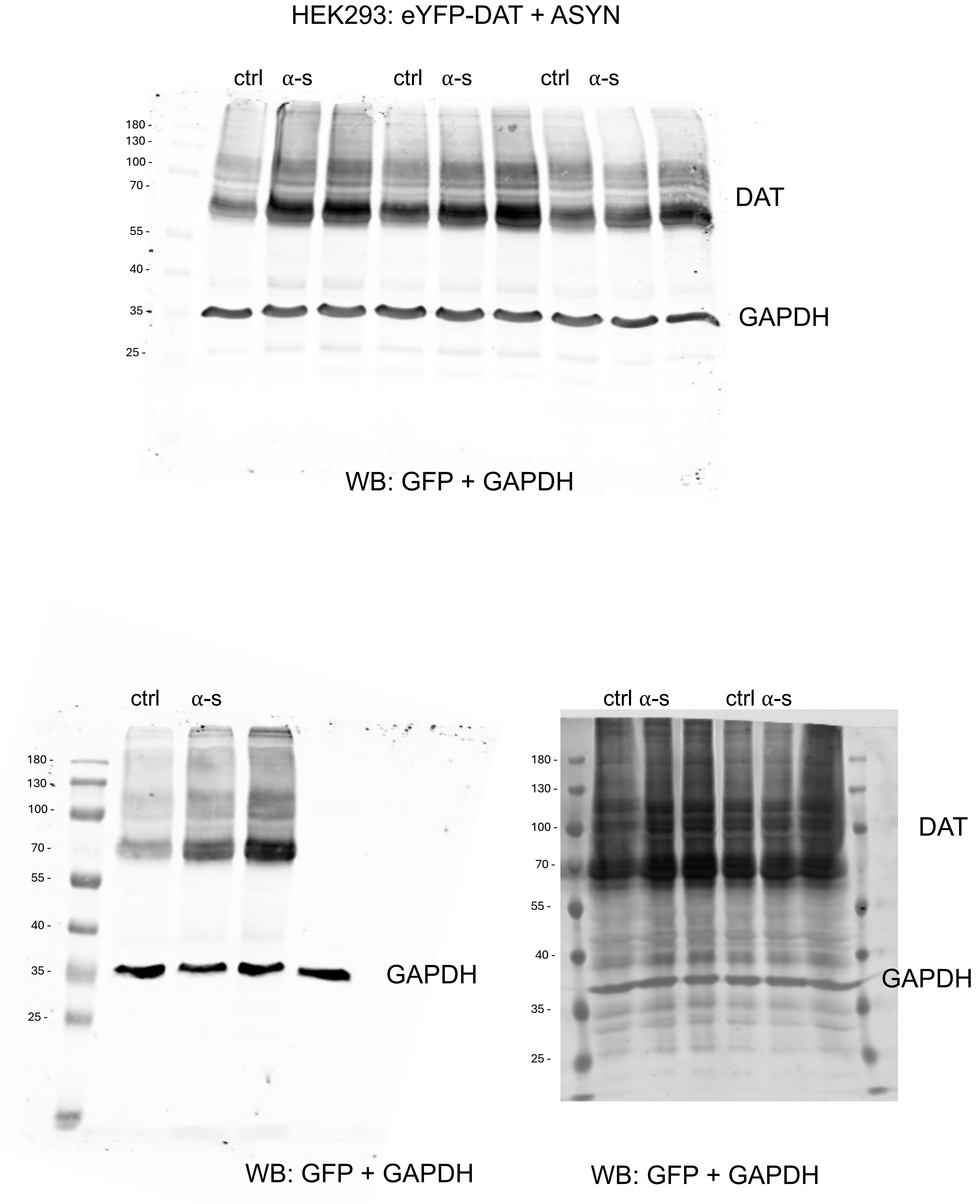

Supplemental Figure 4:

Immunoblots for HEK-293 cells overexpressing α-syn and hSERT.

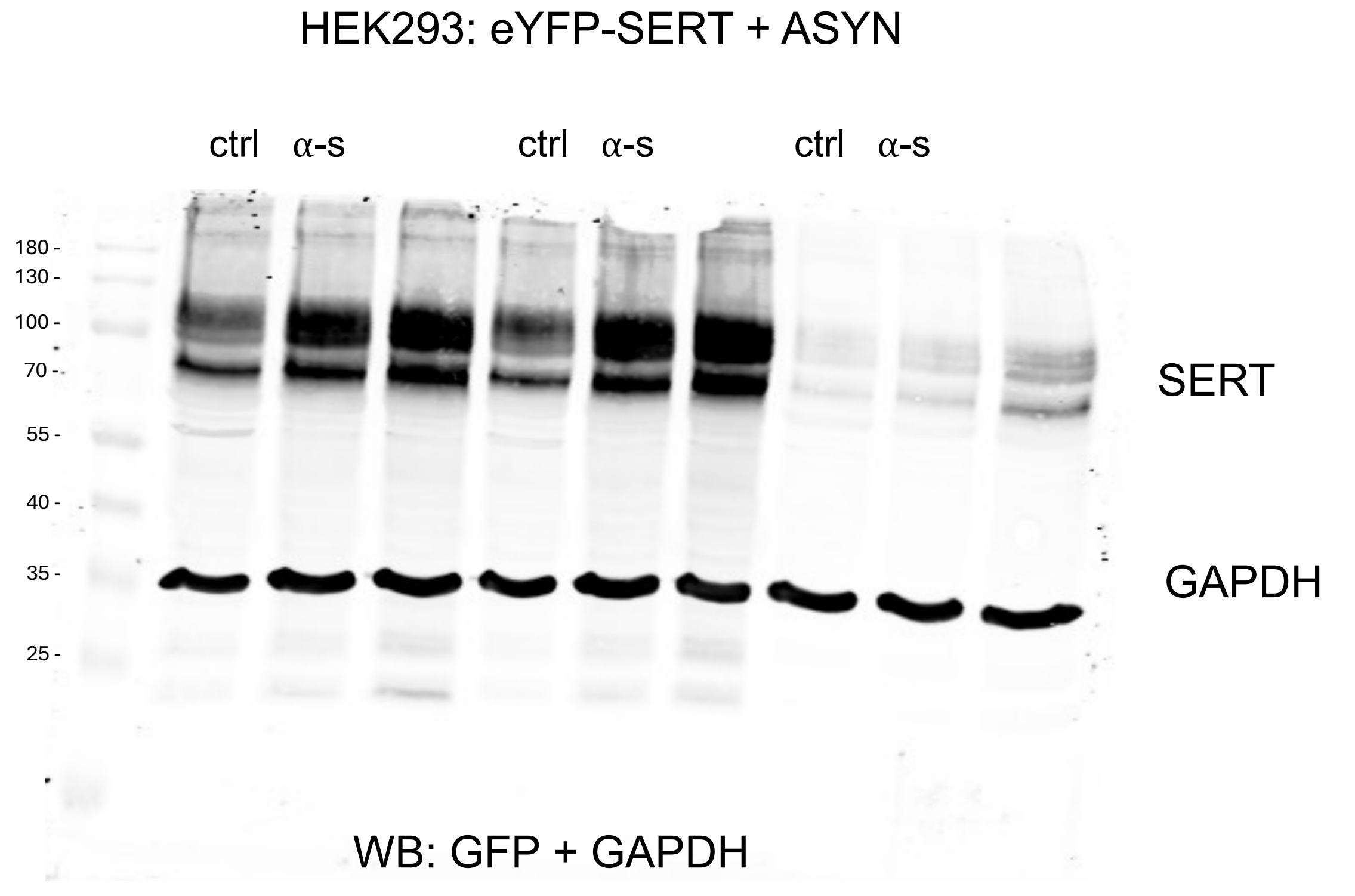

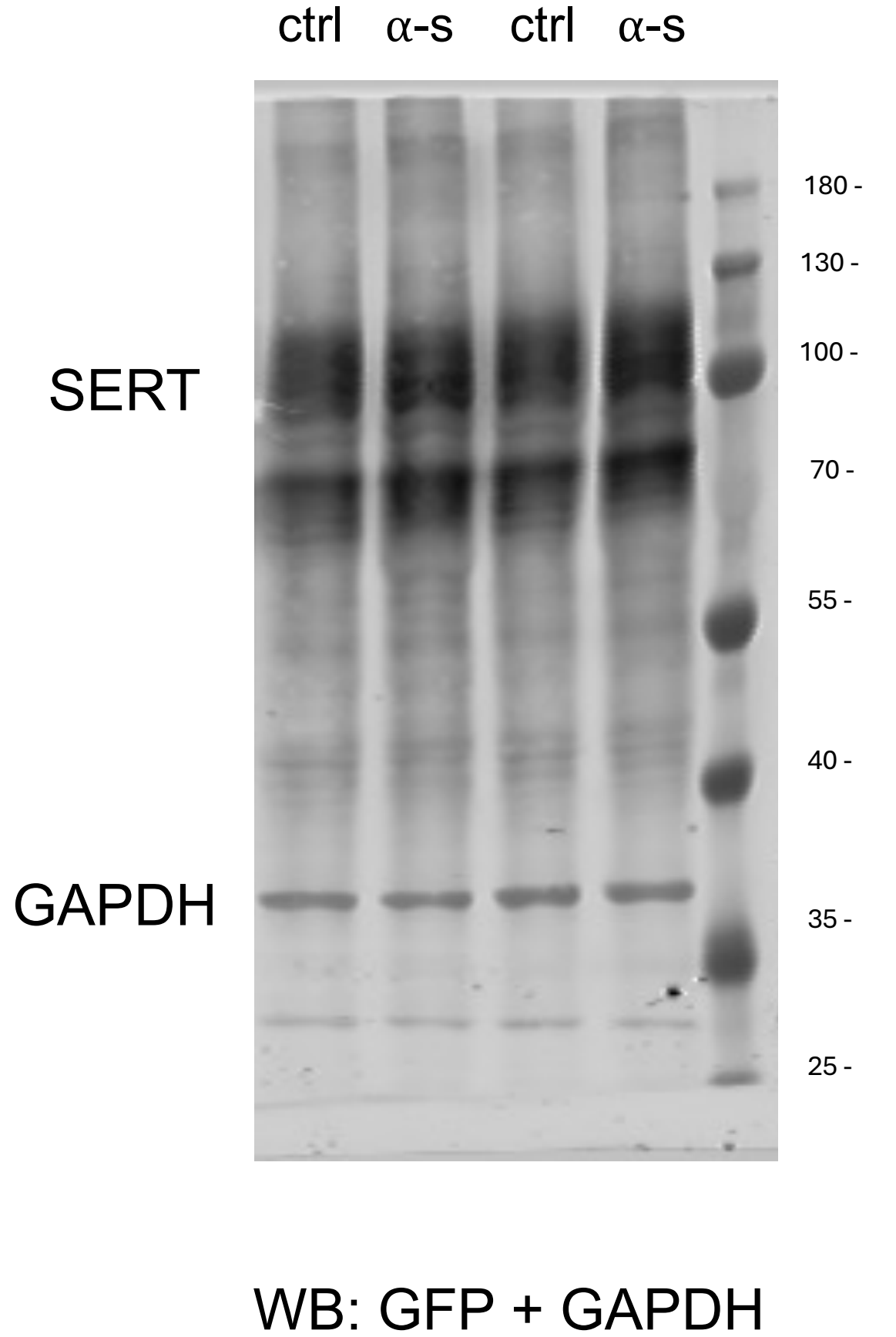

Supplemental Figure 5:

Immunoblots for HEK-293 hDAT cells after siRNA-induced knockdown of α-syn.

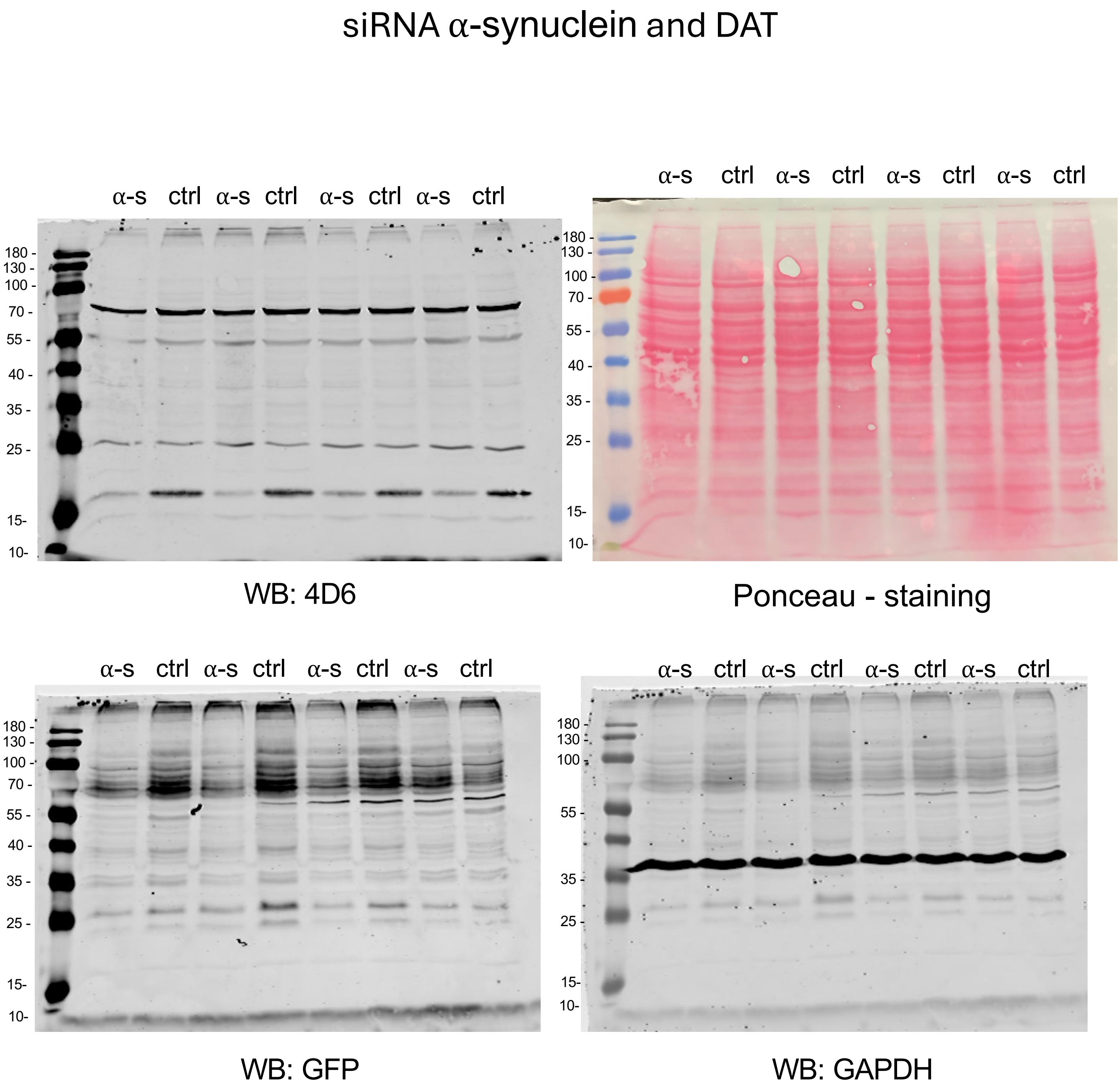

Supplemental Figure 6:

Immunoblots for HEK-293 hSERT cells after siRNA-induced knockdown of α-syn.

siRNA α-synuclein and SERT

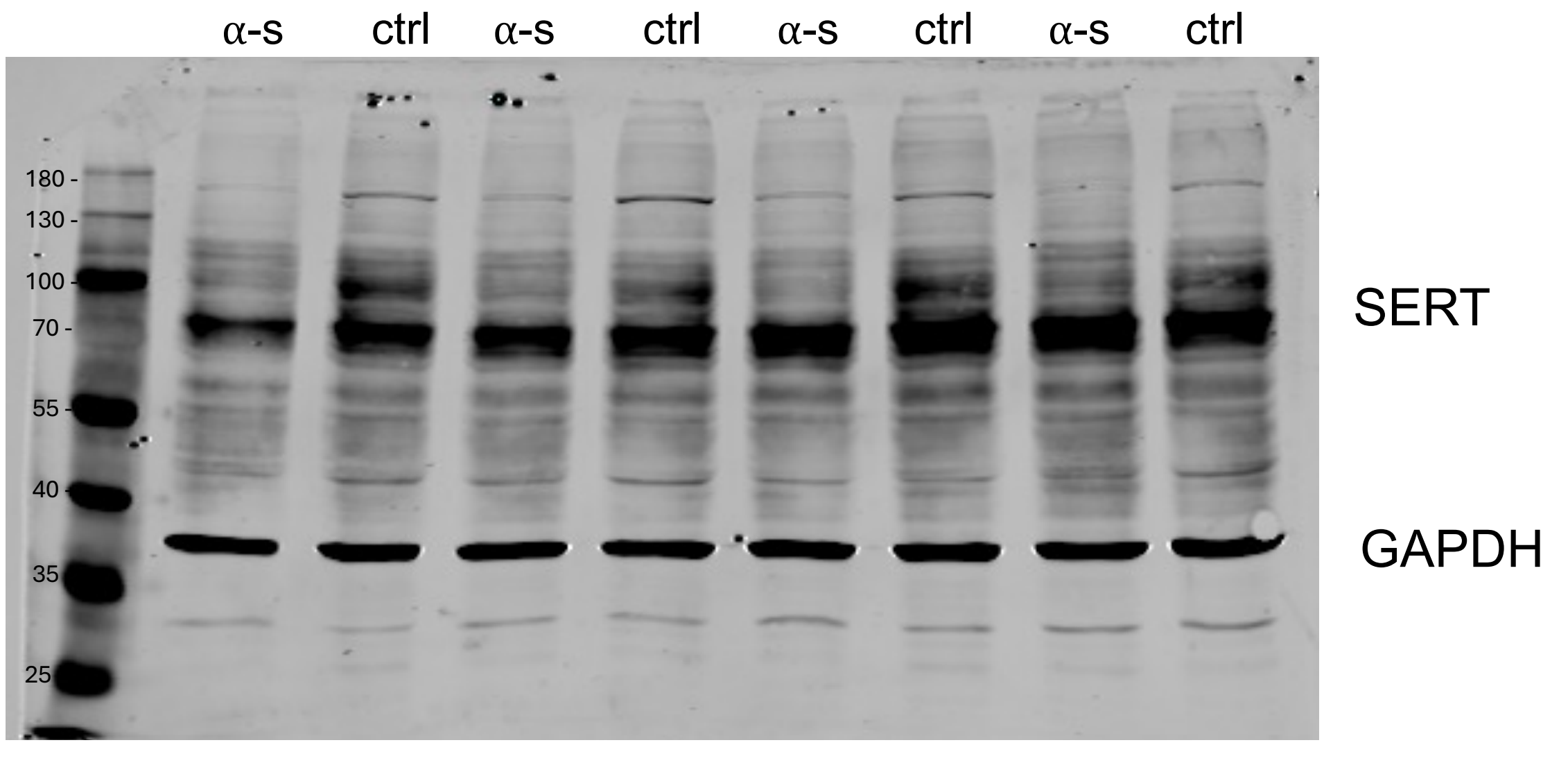

WB: GFP + GAPDH

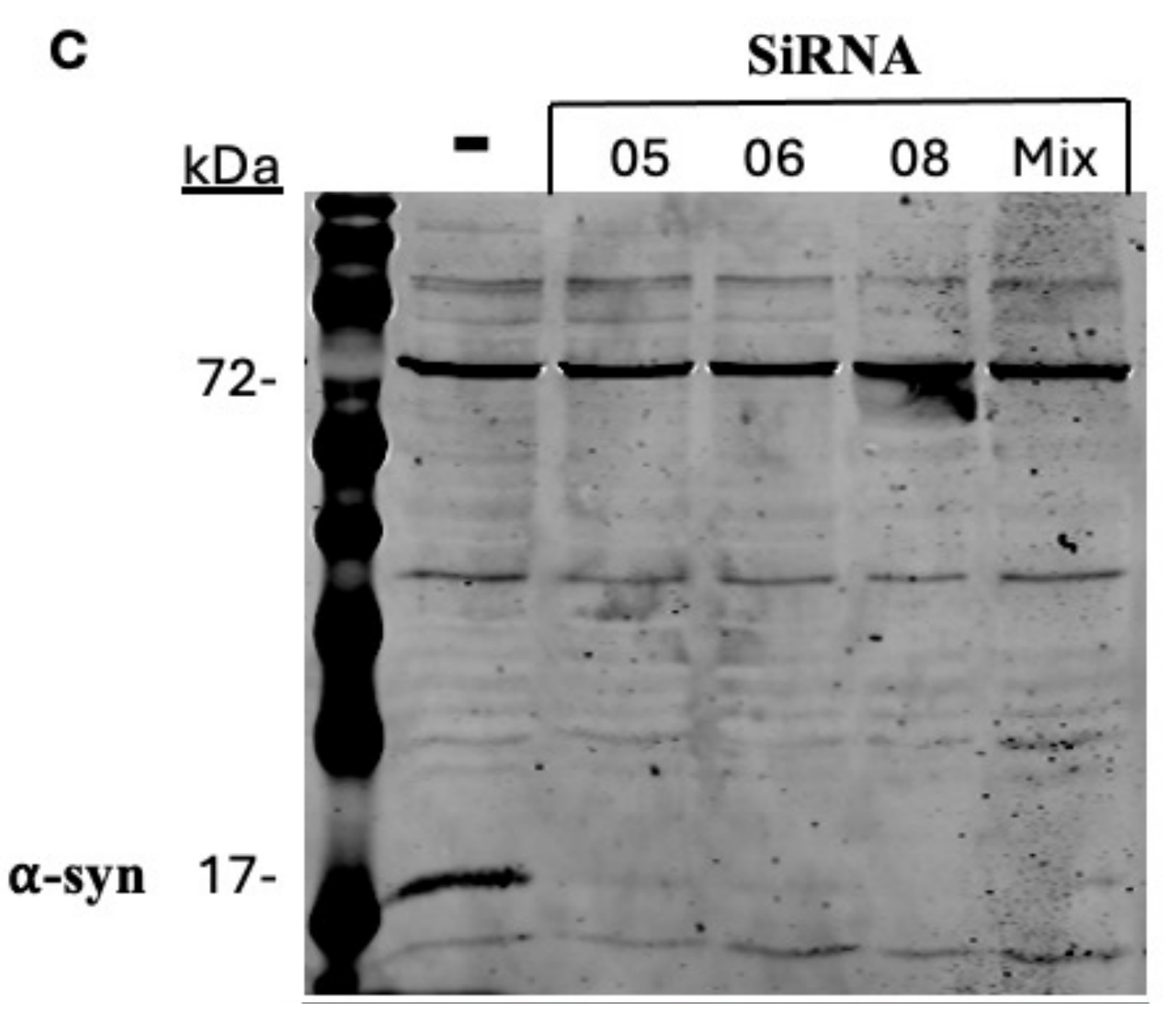

WB: 4D6

Supplemental Figure 7:

Immunoblots for HEK-293 α-syn-KO cells and hDAT.

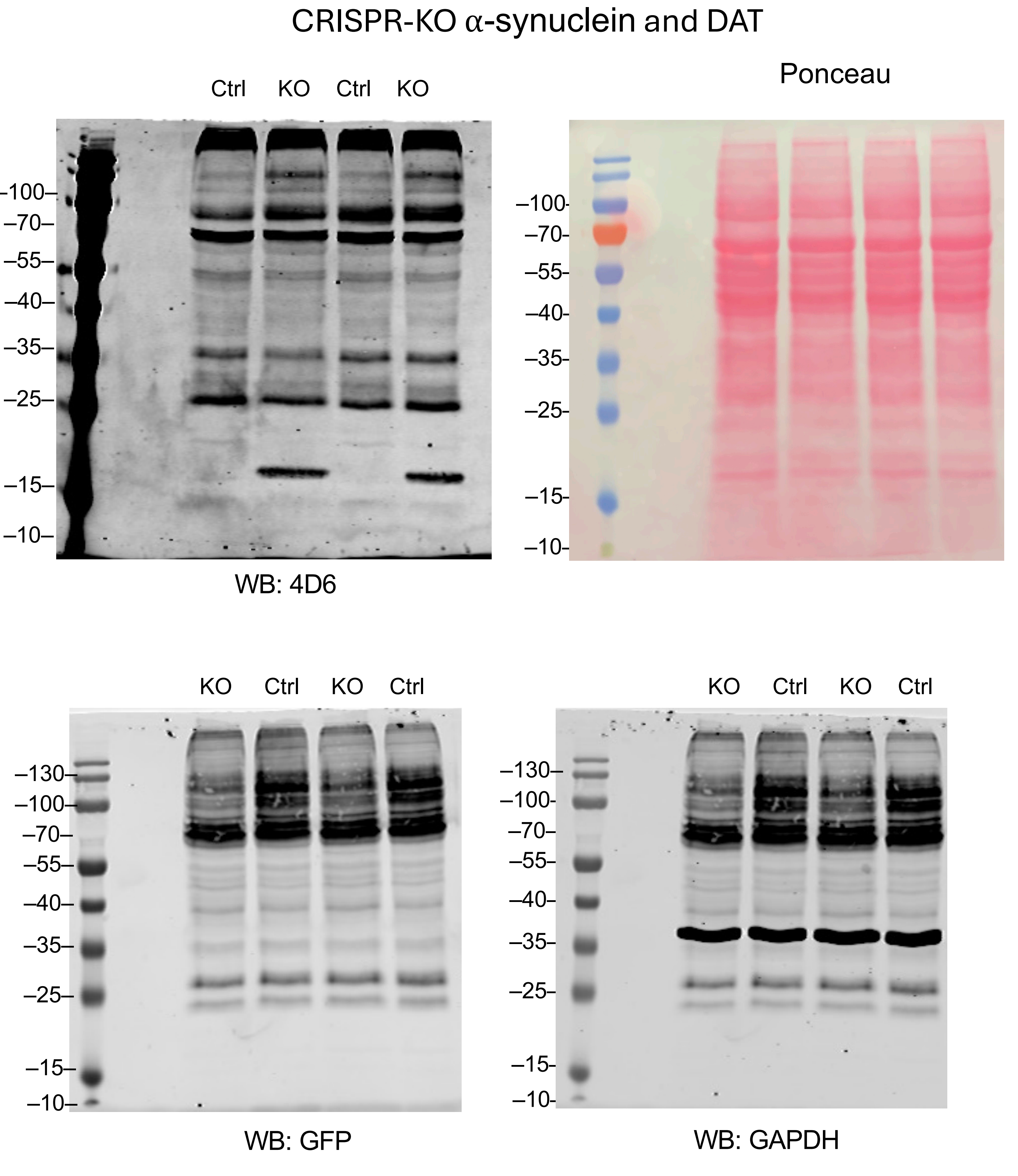

Supplemental Figure 8:
Immunoblots for HEK-293 α-syn-KO cells and hSERT.

CRISPR-KO α-synuclein and SERT

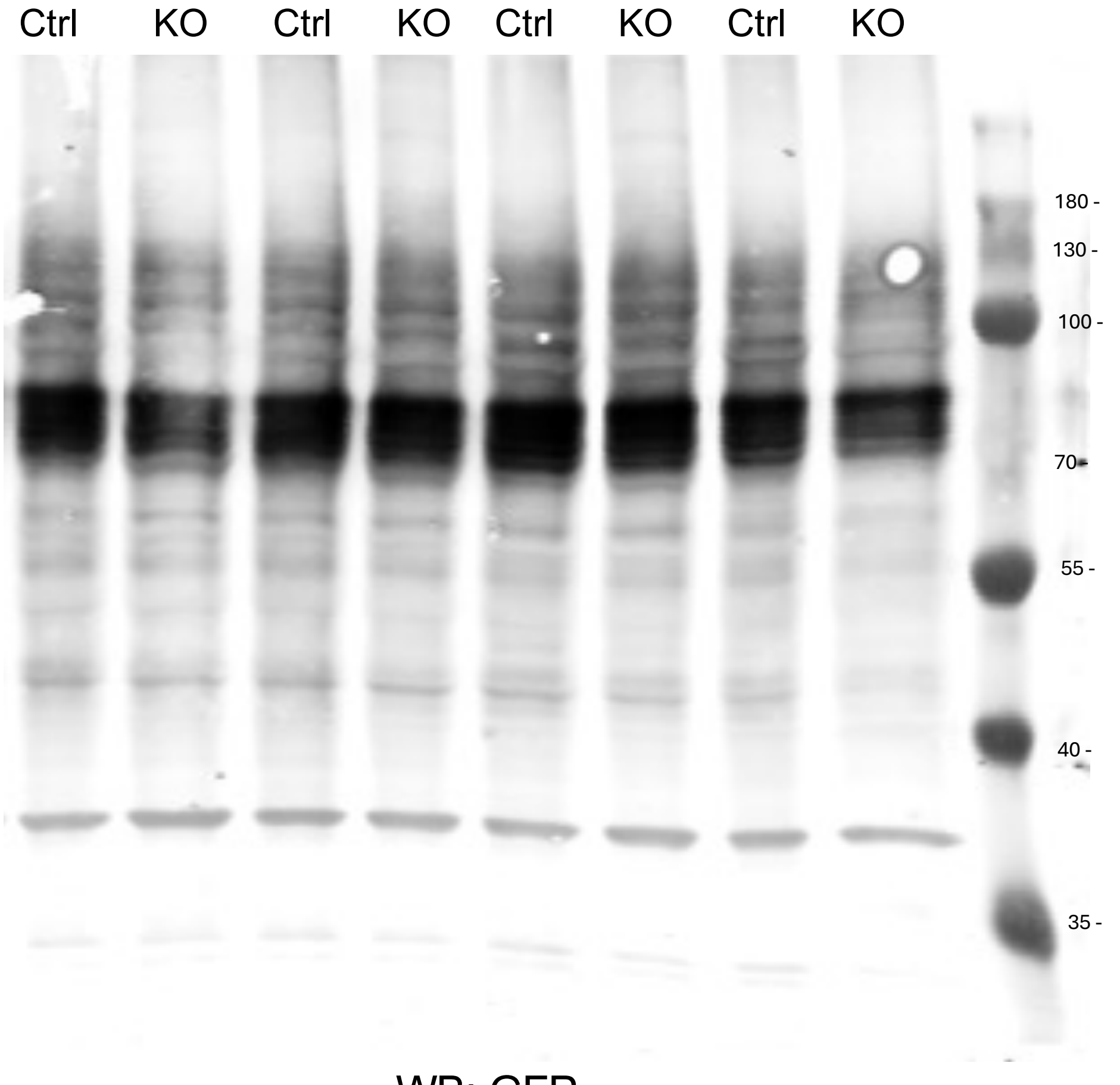

WB: GFP

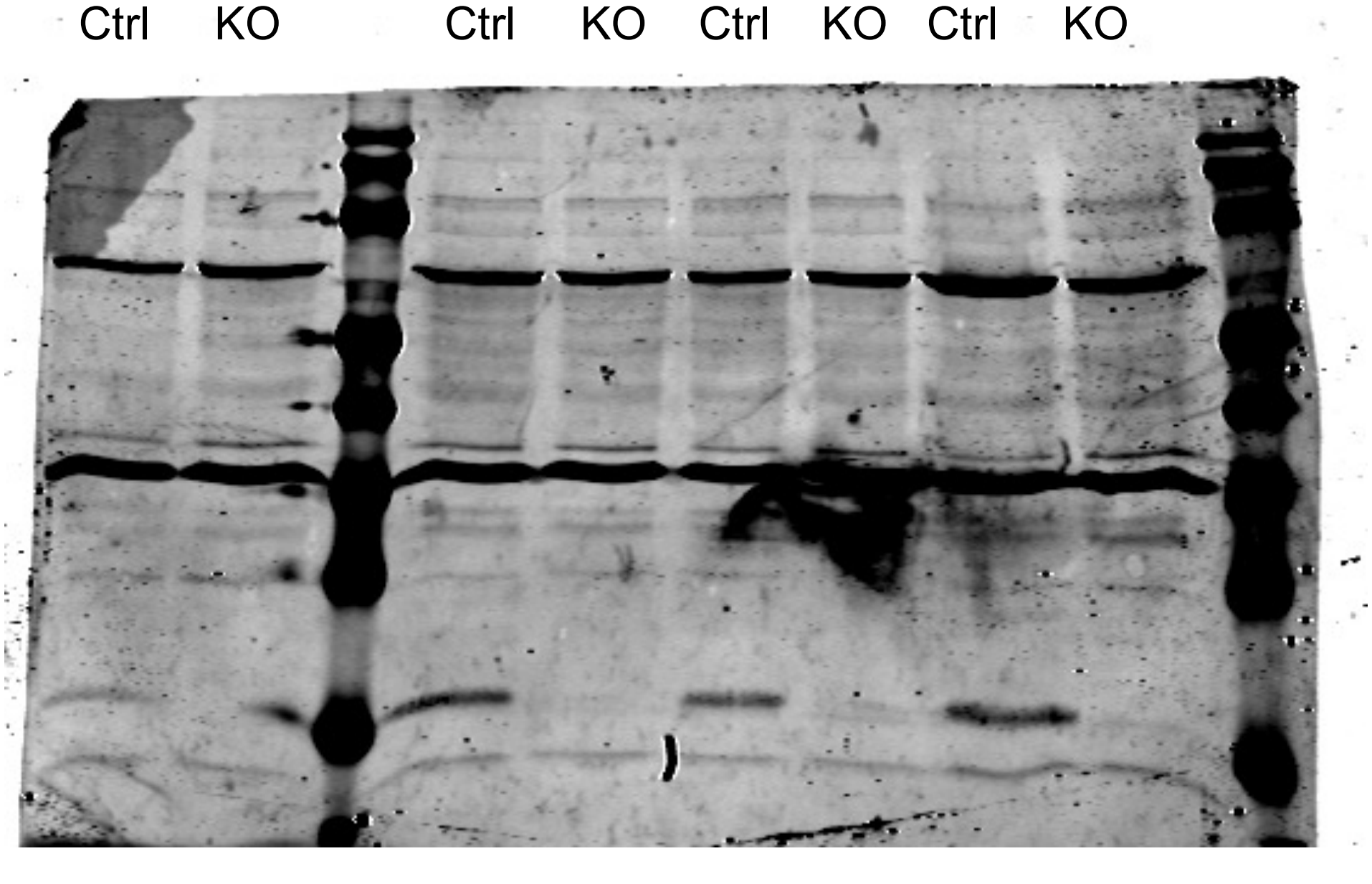

WB: 4D6
